# A Six-Day, Lifestyle-Based Immersion Program Mitigates Cardiovascular Risk Factors and Induces Shifts in Gut Microbiota, Specifically *Lachnospiraceae*, *Ruminococcaceae*, *Faecalibacterium prausnitzii*: A Pilot Study

**DOI:** 10.3390/nu13103459

**Published:** 2021-09-29

**Authors:** Angelica P. Ahrens, Tyler Culpepper, Brittany Saldivar, Stephen Anton, Scott Stoll, Eileen M. Handberg, Ke Xu, Carl Pepine, Eric W. Triplett, Monica Aggarwal

**Affiliations:** 1 Department of Microbiology and Cell Science, College of Agricultural and Life Sciences, University of Florida, 1355 Museum Dr, Gainesville, FL 32603, USA; a.ahrens@ufl.edu (A.P.A.); ewt@ufl.edu (E.W.T.); 2 Department of Medicine, College of Medicine, University of Florida, P.O. Box 100277, Gainesville, FL 32610, USA; tyler.culpepper@medicine.ufl.edu (T.C.); brittsaldivar@ufl.edu (B.S.); 3 Department of Aging and Geriatric Research, University of Florida, 210 E. Mowry Rd, Gainesville, FL 32611, USA; santon@ufl.edu; 4 Total Health Immersions, P.O. Box 741596, Boynton Beach, FL 33474, USA; stollx7@gmail.com; 5 Division of Cardiovascular Medicine, Department of Medicine, University of Florida, 1600 SW Archer Rd, Gainesville, FL 32610, USA; eileen.handberg@medicine.ufl.edu (E.M.H.); carl.pepine@medicine.ufl.edu (C.P.); 6 Department of Health Outcomes and Biomedical Informatics, College of Medicine, University of Florida, 2004 Mowry Rd, Gainesville, FL 32610, USA; xu.ke@ufl.edu

**Keywords:** hypertension, cholesterol, lifestyle, inflammation, whole food, plant based diet, cardiovascular disease, microbiome, hsCRP, TMAO

## Abstract

Cardiovascular disease (CVD) prevalence remains elevated globally. We have previously shown that a one-week lifestyle “immersion program” leads to clinical improvements and sustained improvements in quality of life in moderate to high atherosclerotic CVD (ASCVD) risk individuals. In a subsequent year of this similarly modeled immersion program, we again collected markers of cardiovascular health and, additionally, evaluated intestinal microbiome composition. ASCVD risk volunteers (*n* = 73) completed the one-week “immersion program” involving nutrition (100% plant-based foods), stress management education, and exercise. Anthropometric measurements and CVD risk factors were compared at baseline and post intervention. A subgroup (*n* = 22) provided stool, which we analyzed with 16S rRNA sequencing. We assessed abundance changes within-person, correlated the abundance shifts with clinical changes, and inferred functional pathways using PICRUSt. Reductions in blood pressure, total cholesterol, and triglycerides, were observed without reduction in weight. Significant increases in butyrate producers were detected, including *Lachnospiraceae* and *Oscillospirales*. Within-person, significant shifts in relative abundance (RA) occurred, e.g., increased *Lachnospiraceae* (+58.8% RA, *p* = 0.0002), *Ruminococcaceae* (+82.1%, *p* = 0.0003), *Faecalibacterium prausnitzii* (+54.5%, *p* = 0.002), and diversification and richness. Microbiota changes significantly correlated with body mass index (BMI), blood pressure (BP), cholesterol, high-sensitivity C-reactive protein (hsCRP), glucose, and trimethylamine N-oxide (TMAO) changes. Pairwise decreases were inferred in microbial genes corresponding to cancer, metabolic disease, and amino acid metabolism. This brief lifestyle-based intervention improved lipids and BP and enhanced known butyrate producers, without significant weight loss. These results demonstrate a promising non-pharmacological preventative strategy for improving cardiovascular health.

## 1. Introduction

The prevalence of cardiovascular disease (CVD) remains elevated globally in part due to an increase in cardiovascular risk factors, such as diabetes, hypertension, and hypercholesterolemia [1]. Nearly 50% of adult Americans are now considered hypertensive, [2,3], approximately 12% have elevated cholesterol [4], and almost 11% of patients have diabetes. This increase in cardiovascular risk factors is due, in part, to poor diet, smoking, and sedentary lifestyle [5,6]. The rates of obesity and being overweight now approach 70% of the US population and correlate with a rise in cardiovascular risk factors [7].

Lifestyle-based interventions involving dietary and activity changes have been effective in improving clinical markers of cardiovascular risk, such as body weight and composition, blood pressure (BP), blood glucose, and cholesterol, with concurrent general wellness benefits, such as increased psychological wellbeing [5,8]. Interventions that result in weight loss are associated with significant reductions in known cardiovascular risk factors including BP, low-density lipoproteins (LDL), triglycerides (TG), fasting glucose, and hemoglobin A1c (HbA1c) [9,10,11]. Such lifestyle interventions, whether low or high intensity, have been shown to have lasting effects. For example, a highly controlled, inpatient lifestyle intervention led to a reduction in body weight, blood pressure, glucose, and an increase in HDL, and these effects were sustained for 12 months post-intervention [12]. This program included mandatory physical activity, clinic monitored meals, and group counseling sessions regarding behavior adaptations and coping mechanisms. Low calorie dietary interventions show improvements in atherosclerotic markers in the setting of with weight loss, along with reductions in inflammatory markers (nuclear factor kappa B, NFκB, high-sensitivity C-reactive protein, hsCRP, tumor necrosis factor alpha, TNFα), enhancement of antioxidant systems, and reduction in superoxide production and protein carbonyl groups [13]. Excessive macronutrient load on adipose tissue in obesity promotes inflammation, stimulates monocyte chemoattractant protein-1 (MCP-1), which activates endothelial cells to atherogenic potential. This dysfunction may be an initiator of cardiovascular disease in obesity [14].

In addition to increases in physical activity and calorie restrictions, plant based diets are known to favorably modulate risk factors for cardiovascular disease [15]. Often, it is felt that these diets are beneficial due to their weight reduction leading to improvements in clinical parameters. However, dietary associations with intestinal microbial community composition have been well demonstrated, and it may be through this relationship that dietary changes, outside of weight loss, modulate cardiovascular disease. Accumulating research also indicates that intestinal microbiota community composition is linked to risk of hypertension, obesity, and hyperlipidemia [14,16]. Gut dysbiosis has been shown to contribute to the development and progression of CVD, type 2 diabetes mellitus (T2DM), and some cancers [17]. These bacterial communities are influenced by diet and have effects on immunity and inflammation [18]. For instance, the mucus colonizer *Akkermansia muciniphila* has been shown to attenuate atherosclerosis and promote gut barrier health; treatment can reverse insulin resistance, inflammation in adipose tissue, and metabolic disorders arising from high-fat diet [19]. Butyrate producing bacteria *Roseburia intestinalis* and *Faecalibacterium prausnitzii* are depleted in atheromatous CVD (ASCVD) patients. In addition, *F. prausnitzii* is negatively correlated with uric acid levels in serum, which has been noted to be elevated in a diet high in red meat [20]. Intestinal microbiota also make trimethylamine (TMA) in response to foods high in choline, such as red meat, eggs, and dairy, and then is oxidized in the liver to trimethylamine-N-oxide (TMAO). The role of TMAO in human health is controversial. Previous data have associated elevated TMAO with cardiovascular events, possibly due to effects on the arterial wall and/or platelet reactivity by secreting microbial metabolites [21,22,23]. However, the anti-oxidant rich Mediterranean diet, rich in seafood, which carries naturally occurring TMAO, has proven benefit in preventing or attenuating cardiometabolic disease. Some recent data in animal models suggest that other similar molecules, such as TMA, may play more of a role in cardiovascular health than TMAO [24,25,26]. Future studies are needed.

Few studies have shown a linkage between plant-based diets, changes in gut microbial community composition, and markers of cardiovascular health in the same setting. One such study demonstrated that a plant-based diet was effective in both modifying gut microbial communities and improving markers of cardiovascular health but failed to show the specific relationships between bacterial groups and individual taxa with particular markers of cardiovascular health [27]. Such findings suggest that dietary changes resulting in improved cardiovascular outcomes may be associated with changes in intestinal microbiota, but the underlying mechanisms are yet to be discovered.

We previously demonstrated in a pilot study that a short, one week lifestyle intervention involving changes in both diet (with a focus on plant-based foods) and physical activity significantly lowered blood pressure, total cholesterol, triglycerides and LDL cholesterol [28]. In that study, among participants with HTN (71%) at baseline, both DBP and SBP declined after the intervention. That intervention also resulted in significant body weight reduction, so it was not clear if the observed effects were due to dietary changes or the weight loss. Questions arose whether these results could be reproduced and whether there were other possible mechanisms for the clinical changes other than weight loss. Accordingly, we observed a similar intervention on an independent cohort and again assessed its effects on markers of cardiovascular health. Furthermore, we investigated changes in gut microbiota community composition and their associations with markers of cardiovascular health. Here we present a preliminary analysis of the changes in serum markers of cardiovascular health, gut microbiota community composition, and their associations before and after the intervention.

## 2. Materials and Methods

**Figure 1:**
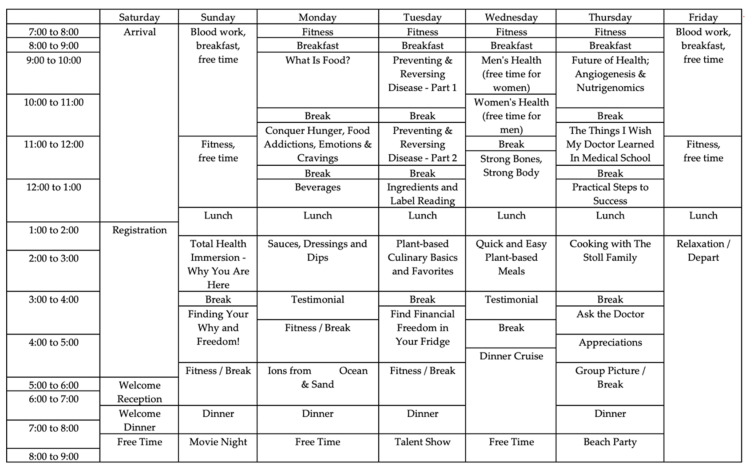
Schedule of the six-day intervention.

For the trial, 73 adults (37 male, 36 female) voluntarily applied to participate in a one-week, in-house, lifestyle-based “Immersion Program” with a significant other, in the fall of 2019. This program included daily nutrition education; meals that were 100% whole food; plant-based foods (WFPB) with minimal sugar, salt, and oil; cardiopulmonary exercise; and stress management classes. A detailed menu of foods that were made available to the participants is provided in Supplementary Material. Participants were encouraged to consume all provided foods, which constituted three meals per day, and take part in daily exercise and yoga classes although these were not required. There was no caloric or quantity restriction. They attended optional educational lectures on nutrition for 2–5 h each day, which were organized and primarily delivered by a physician team. Participants were encouraged to stay in the provided hotel room. During the welcome dinner, the research project was discussed. The itinerary for the program is provided in Figure 1. The Immersion Education Program involved 15 h of nutritional education, five hours of dedicated fitness (with three additional hours of sports/games such as relay races, kick ball, and volleyball), two hours of stress reduction education, and four 1-hr cooking demonstrations.

On day 0 of the immersion (T1), participants provided written informed consent for the research. The study was approved by University of Florida’s Institutional Review Board IRB201901936. Study data were collected and managed using Research Electronic Data Capture (REDCap) electronic data capture tools hosted by the Clinical and Translational Science Institute (CTSI) at the University of Florida [29].

Anonymized data and materials have been made publicly available at the NCBI BioProject (accession number PRJNA766548) and can be accessed at https://www.ncbi.nlm.nih.gov/bioproject/PRJNA766548. The trial was registered at Clinicaltrials.gov, NCT03320551 on 25 October 2017. Funding for the trial was provided by Whole Foods, Inc. (Austin, TX, USA) and Total Health Immersions, Inc. (Boynton Beach, FL, USA).

### 2.1. Anthropometric and Cardiovascular Measures

Anthropometric measurements, blood pressure, serum labs, and stools samples were collected on days 0 and 6 (T2). The heights and weights of the participants were obtained using a stadiometer and a digital sale. Hip and waist circumferences were obtained via tape measure. Systolic (SBP) and diastolic blood pressure (DBP) were measured by physicians or nurses using an automatic blood pressure cuff. Fasting plasma labs were obtained via venipuncture. Plasma lipids, blood sugars and high-sensitivity C-reactive protein (hsCRP) collection and analyses were performed by LabCorp. Plasma samples for trimethylamine N-oxide (TMAO) analysis were centrifuged, chilled, and provided to Cleveland Heart labs for analysis.

### 2.2. Gut Microbiome

Participants collected their stool samples with a stool collection kit and placed the sample on ice in a cooler at the study site. Within twelve hours, these samples were transferred to a −80 °C freezer. It was demonstrated that microbial communities in fecal samples do not change if the samples are frozen within 24 h of collection [30].

Bacterial DNA was extracted from 47 fecal samples collected at T1 (*N* = 24) and at T2 (*N* = 23) using the E.Z.N.A Stool Extraction kit following the manufacturer’s protocol (Omega Bio-Tek, Doraville, CA, USA). The V3–V4 variable region of the 16S rRNA gene was polymerase chain reaction-amplified using a universal primer set (341F and 806R primers) with unique barcodes for each sample, purified with Qubit dsDNA High Sensitivity (Invitrogen, Life technologies Inc., Carlsbad, CA, USA), and quantified with the 1X dsDNA High Sensitivity kit with a Qubit 2.0 fluorometer (Invitrogen, Life Technologies Inc., Carlsbad, CA, USA). Equal masses of amplicons (40 ng DNA) were pooled for sequencing with unique barcodes and sequenced at UF Interdisciplinary Center for Biotechnology Research (ICBR) using the Illumina MiSeq platform (2 × 300 paired end reads) according to the manufacturer’s protocol (ICBR, Gainesville, FL, USA), as we have done previously [31,32].

Raw reads were joined and demultiplexed using Qiime1 [33]. Amplicons were filtered and trimmed using Divisive Amplicon Denoising Algorithm (DADA2) [34] version 1.11.3, which yields amplicon sequence variants (ASVs) with single nucleotide resolution for bacterial marker-gene analysis. Primers were removed and reads filtered and trimmed to 400 bases with these parameters maxN 0, truncQ 11, and maxEE 1. Chimeric sequences were removed. Each ASV was classified by a naïve Bayesian classifier method available in DADA2, using the Silva 138 database [35].

Samples were rarefied to 13,000 reads to normalize sequencing depth using the phyloseq [36] R package, and raw counts were transformed to relative abundance. ASVs were aggregated to higher taxonomic classifications as appropriate. For alpha diversity calculations inspecting within person change, rarefaction excluded the three samples from subjects who did not have microbiome data at both timepoints and was thus applied at a minimum library size of 112,133 read counts.

Medications were assessed and none of the participants who provided stool samples was on antibiotics at the time of the study.

### 2.3. Data Analyses

Changes in clinical variables were evaluated using paired *t*-tests in SPSS version 26 (IBM). The Benjamini-Hochberg procedure was used to correct for multiple comparisons. Participants were categorized into one of four subgroups based on blood pressure response over time (HTN at baseline and at final, HTN at baseline and normotensive at final, normotensive at baseline, and HTN at final, and normotensive at both baseline and final) and χ2 tests were performed to assess for changes.

A total of 4305 unique bacterial sequences, or ASVs, were identified across the 47 samples collected at either T1 or T2, with 2114 ASVs having a raw count of at least 2. 9,195,143 total reads were sequenced across these samples, with an average of 195,641 reads per sample and a maximum of 287,236 reads observed. After rarefaction, 3155 ASVs remained across the 47 samples, representing 16 phyla, 26 classes, 56 orders, 86 families, 234 genera, and 218 species. Of the 4305 distinct ASVs observed, only 745 ASVs were present in at least three individuals at T2 and only 312 of the ASVs were present in at least five individuals.

Linear discriminant analysis (LDA) was performed as a first pass in high-dimensional biomarker discovery for taxa distinct across the timepoints, using linear discriminant analysis effect size (LEfSe [37]) in Nephele [38]. Alpha diversity measures of richness and evenness (ACE, Chao1, Shannon, Simpson, Fisher) were calculated in MicrobiomeAnalyst [39] on the dataset comprised of 44 subjects with both T1 and T2 samples, rarefied to the minimum library size of 112,133.

Wilcoxon paired tests were performed in SPSS on alpha diversity measures as well as on relative abundances of the microbiota at the family, genus, species, and ASV-level for within-person changes in the gut microbiota. To identify significant shifts that occurred in the gut milieu *within person*, at the family, genus, and species-level, the top 20 taxa were identified at each timepoint, based on their median relative abundance. Pairwise Wilcoxon tests were then conducted for each taxon to assess the degree of change in relative abundance, for a total of 23 families, 23 genera, 27 species, and 24 ASVs. Spearman correlations were performed to ascertain whether changes in the relative abundance of any of the most abundant microbes were significantly associated with change in the clinical measures. This analysis was also carried out at the family, genus, species, and ASV levels. Spearman correlations were calculated in R to assess correlations of microbiota changes with changes in the clinical measures across the intervention, with FDR adjustments of statistical significance.

Comparisons across clinical groups were performed in R with differential expression analysis based on the negative binomial (a.k.a. Gamma–Poisson) distribution (DESeq2) [40], prevalence interval for microbiome evaluation (PIME) [41], and non-parametric tests. We categorized participants according to these guidelines: as patients with hsCRP levels ≥ 2 mg/L have been shown to be at higher risk for major adverse cardiovascular events [42], we defined <2 mg/L as the cut-off for low hsCRP in this study. Participants with BP less than 120/80 mm Hg were designated as having “normal” BP, and those with SBP ≥ 120 or DBP ≥ 80 designated as “elevated or hypertensive”, according to the American College of Cardiology and the American Heart Association (ACC/AHA) guidelines [2,43]. LDL levels within 110 were designated as “desirable.”

To identify differentially abundant genera based on BP, LDL, and hsCRP at T2, we conducted differential expression analysis based on the negative binomial distribution on the raw counts, using normalization factors to account for library depth differences in dispersion estimates with generalized linear model fitting (DESeq2 R package) [40]. With the Prevalence Interval for Microbiome Evaluation (PIME) [41] R package we calculated the prevalence interval at which the clearest separation in microbial community composition was observed between those who exhibited low versus high hsCRP at T2 and the lowest out of bag (OOB) error rate achieved. At this interval, we identified the top 29 ASVs with the highest mean decrease accuracy (MDA) which contributed to the random forest classification. Bacterial gene content from the 16S rRNA sequencing data was estimated with Phylogenetic Investigation of Communities by Reconstruction of Unobserved States (PICRUSt) [44] via Nephele [38]. We assessed differences in predicted functional content related to metabolic pathways and bacterial genes implicated in human disease with a Wilcoxon paired test across timepoints.

Using the MicrobiomeAnalyst [39] pipeline, classical univariate analyses (Mann-Whitney U tests) were performed to identify the ASVs, species, and genera significantly differentially abundant with respect to hsCRP or TMAO status at T2. For this analysis, features with identical counts (e.g., zero) and that appeared exclusively in one sample were considered artifacts and were excluded. Low count and low variance filters removed low quality features with either <10% of its values containing at least 4 counts or within the lowest 5% inter-quartile range (IQR) suggesting that its variance remained close to constant. A total of 1344 low abundance features and 39 low variance features were removed based on prevalence and IQR, respectively, resulting in a filtered dataset of 731 features. Data were rarefied to the minimum library size, or to 112,133 for analyses strictly limited to T2, and subjected to a centered log ratio (CLR) transformation to enable biologically meaningful comparative analysis.

## 3. Results

### 3.1. Anthropometric and Cardiovascular Measures

A total of 73 participants completed the immersion program: mean age 46.89 years (+/− 12.38), mean BMI 31.14 (+/− 8.83), 60.7% were women, 76.7% had HTN at day 0. Pertinent demographic and anthropometric data are summarized in Table 1. After the intervention, minor but significant increases were observed for BMI (0.47 kg/m^2^ +/− 0.39, *p* < 0.0001) and body weight (1.35 kg +/− 31.86, *p* < 0.0001). Significant decreases were observed for waist circumference (−2.69 cm +/−21/06, *p* = 0.0408) and hip circumference (−1.09 cm +/−18.54, *p* = 0.0372).

Despite the brief duration of the lifestyle intervention program, significant changes were noted in SBP and DBP as well as in total cholesterol (TC), TG, and LDL, whereas minor but significant decreases were observed for high-density lipoprotein (HDL). Neither hsCRP nor TMAO demonstrated significant change after one week.

At baseline, 56 participants had HTN. Demographic and clinical variables based on absence or presence of HTN appear in Table 2. Eleven participants who were hypertensive at day 0 decreased their BP to the normal range of <130/80 mmHg (mean SBP change: −10.45 ± 7.55 mmHg (*p* = 0.026), mean DBP change: −9.64 ± 7.26 mmHg (*p* = 0.001)). In subjects who took BP lowering medication versus those who did not, there were no significant changes in any clinical variables. When participants were categorized into groups based on either TMAO or hsCRP changes (i.e., whether they experienced an increase versus a decrease), no significant differences in changes of any other clinical variables were observed (data not shown).

### 3.2. Gut Microbiome

**Figure 2:**
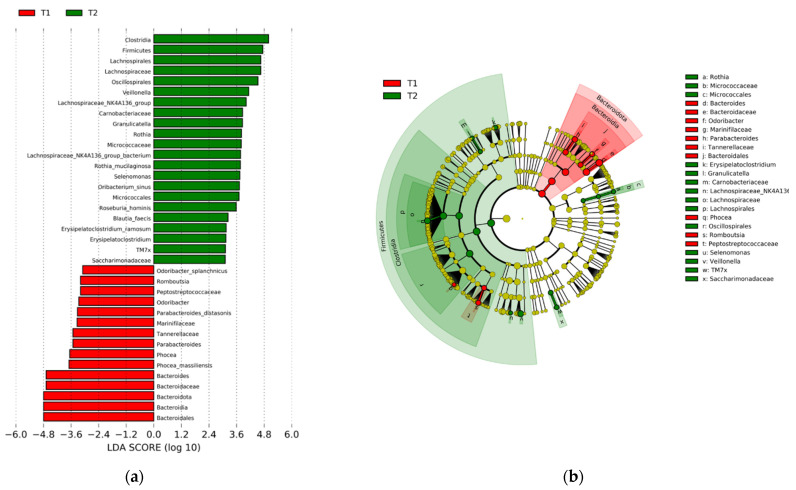
Microbial shifts across the intervention among the most abundant taxa. (**a**) Bacterial taxa significantly increased in subjects at T1 (red) versus T2 (green) as identified by **Linear discriminant analysis Effect Size** (LEfSe) analysis; (**b**) bacterial clades of the respective bacteria.

Gut microbiome diversity increased significantly over the six-day intervention as per the Simpson (T1 median ± SD 0.93 ± 0.07, versus T2 0.94 ± 0.02, z = −2.68, *p* = 0.007) and Shannon diversity (T1 3.62 ± 0.49, vs. T2 median 3.77 ± 0.29, z = −2.94, *p* = 0.003) indices. Observed richness (estimate of number of unique ASVs in each sample, *p* = 0.126), ACE (*p* = 0.116), Chao1 (*p* = 0.089), and Fisher (*p* = 0.124) also increased within-person, although not at *p* < 0.05. Overall, an increase in butyrate producers, particularly those in the Clostridia class, was observed in the samples across T1 and T2, at the expense of Bacteroidia (Figure 2). No significant differences were observed in the baseline characteristics of the subgroup providing stool samples versus the full cohort.

Of the most abundant taxa identified at each timepoint, significant differences within individual were observed in 16 families, 16 genera, 18 species, and 17 amplicon sequence variants (ASVs). Statistics and median relative abundances for the significant Wilcoxon tests are provided in Table 3. Nonsignificant findings and full statistics for all tests are provided in Supplementary Table S1. The greatest decreases within individual were observed in the *Bacteroides* genus of *Bacteroidaceae* (−15.75%) and the *Phascolarctobacterium* genus of *Acidaminococcaceae* (−10.88%). The greatest increases were observed in *Lachnospiraceae* (10.30%), specifically the *Roseburia*, *Lachnospiraceae NK4A136 group*, *Blautia*, and *Anaerostipes* genera, and *Ruminococcaceae* (7.65%), specifically the *Faecalibacterium*, *Subdoligranulum*, and *Ruminococcus* genera (Table 3).

The greatest increase at the species level was observed in *Faecalibacterium prausnitzii*, and the greatest decrease observed in *Bacteroides vulgatus*, each with two associated ASVs. The significant increase in *Veillonella dispar* is partially responsible for the increase observed in the genus. Other species that significantly increased were *Blautia obeum*, *Anaerostipes hadrus*, *Lachnospiraceae NK4A136 group bacterium*, *Adlercreutzia equolifaciens*, *Roseburia hominis*, *Blautia faecis*, *Bacteroides thetaiotaomicron*, and *Dorea formicigenerans*. Those that significantly decreased within-person were *Blautia massiliensis*, *Alistipes putredinis*, *Parabacteroides distasonis*, *Parabacteroides merdae*, *Bacteroides caccae*, *Bilophila wadsworthia*, and *Coprococcus comes*. Differences in several of these species may be driven by a select few of the most abundant ASVs. The sequences for these ASVs are provided in Supplementary Material Table S2.

#### 3.2.1. Bacterial Families and Genera Correlated with Clinical Measures

**Figure 3:**
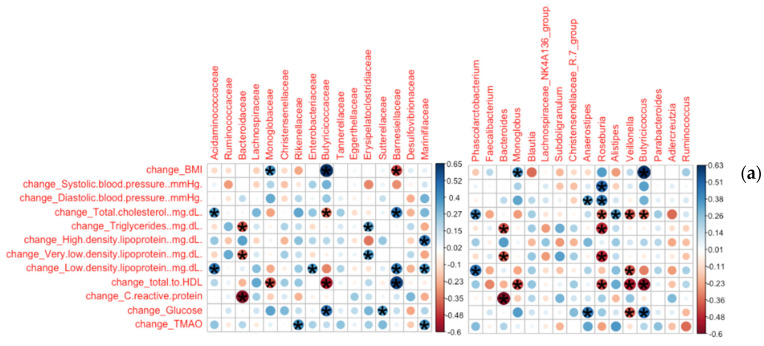

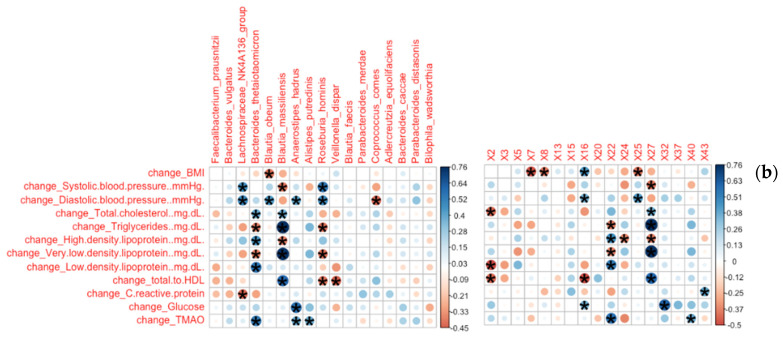
Change in relative abundances correlated with change in anthropometric measures. Spearman correlation of change in relative abundance and change in anthropometric measures across the intervention. Significant correlations are marked with an asterisk, with color and size of the circle indicative of the strength of correlation at right. (**a**) Family and genus level correlations; (**b**) species and ASV level correlations. ASV sequence IDs indicated by X. BMI, body mass index; HDL, high-density lipoprotein; TMAO, trimethylamine N-oxide.

Changes in the relative abundance of *Roseburia* of *Lachnospiraceae* were positively associated with changes in both systolic and diastolic blood pressure (Figure 3). Change in *Anaerostipes* also positively associated with diastolic blood pressure change. *Bacteroides* changes were negatively associated with changes in triglycerides and VLDL, as was change in *Roseburia*, while *Erysipelatoclostridiaceae* change positively associated with changes in those variables. LDL change was positively associated with changes in *Phascolarctobacterium*, *Enterobacteriaceae*, *Barnesiellaceae*, and *Marinifilaceae* and negatively associated with change in *Veillonella*. Change in total cholesterol to HDL ratio negatively correlated with changes in *Roseburia*, *Veillonella*, and *Butyricicoccus*, and positively correlated with change in *Barnesiellaceae*.

#### 3.2.2. Bacterial Species and ASVs Correlated with Clinical Measures

The change in relative abundance in *Blautia massiliensis* was related to a significant change in triglycerides and VLDL (ρ = 0.76, p_FDR_ = 0.0019). Change in *Anaerostipes hadrus* was positively associated with a significant change in glucose levels. Change in systolic blood pressure was positively associated with a significant change in *Collinsella aerofaciens* (ρ = −0.64, p_FDR_ = 0.042) and inversely associated with change in *Bacteroides uniformis* (ρ = −0.62, *p* = 0.052).

The greatest decreases in three ASVs, ASV 7, ASV 8, and ASV 25, each representing a strain of *B. uniformis*, *B. vulgatus*, or *B. obeum*, respectively, occurred in those individuals with the greatest decreases in BMI, while ASV 16, belonging to *Monoglobus*, increased. This ASV was also positively correlated with changes in diastolic blood pressure and glucose and negatively associated with change in total:HDL ratio. ASV 2, belonging to *Faecalibacterium prausnitzii*, was the only feature at this resolution negatively correlated with total cholesterol and LDL, as well as with change in total:HDL ratio.

ASV 22 of *B. thetaiotaomicron* exhibited the strongest, positive correlation with change in TMAO. ASV 40 (*Alistipes putredinis*) also positively associated with TMAO change. Two ASVs, ASV 24 (Subdoligranulum) and ASV 27 (*Blautia massiliensis*) negatively associated with change in HDL, the latter of which also exhibited the strongest positive correlation with change in triglycerides and VLDL. ASV 32 (*Anaerostipes hadrus*) exhibited the strongest positive correlation with change in glucose, and ASV 43 belonging to *Subdoligranulum* positively associated with change in hsCRP.

#### 3.2.3. Microbiota Are Differentially Abundant across Clinical Subgroups of Hypertension, LDL, and hsCRP at T2

Sequences belonging to the family *Akkermansiaceae* as well as the genus *Akkermansia* were found at higher abundance in individuals with a desirable LDL (log2fold change of −8.91 and −8.84, respectively; Table 4). The species *Akkermansia muciniphila* was increased (9.43 log2fold change) in individuals with low hsCRP, as well as *Lactobacillus paracasei* (log2fold change of 7.00) and members of the *Lactobacillus* genus (log2fold change of 4.76) and *Lactobacillaceae* family (log2fold change of 4.82). *Phascolarctobacterium* and *Phascolarctobacterium faecium* increased in individuals with a desirable LDL (log2fold change −7.37 and −8.28, respectively). On the contrary, two species of *Prevotella*, *Prevotella copri,* and an undefined *Prevotella* increased in individuals with high LDL, while *Romboutsia* and *Romboutsia ilealis* increased in those with hypertension. The species *Alistipes onderdonkii* was increased in both the high hsCRP and high LDL subgroups independently (log2fold change −4.65 and 5.61, respectively).

Furthermore, microbiota prevalence could correctly classify individuals with a high hsCRP (*N* = 10) vs. those with a low hsCRP (*N* = 13) at T2 by random forest. Prior to filtering by prevalence interval, the OOB error rate for hsCRP level at T2 was 0.3913, which was reduced to 0.0435 after filtering at the optimal prevalence level of 40%. At this prevalence interval, 240 of the ASVs remained (Supplementary Figure S1). Of the ASVs that contributed most significantly to the random forest model, 33.3% were classified as *Lachnospiraceae*, 20% as *Oscillospirales*, 10% as *Bacteroidaceae,* and 10% as *Ruminococcaceae* (sequences available in Supplementary Table S3). Statistics and taxonomy are provided in Supplementary Table S4 for this analysis.

#### 3.2.4. Functional Predictions from the 16S Data Suggest a Downregulation of Bacterial Genes Implicated in Metabolism of Amino Acids, Carbohydrates, and Glycans, as Well as Human Cancer and Metabolic Disease

PICRUSt functional predictions estimated the most prevalent genes were those belonging to membrane transport, cellular processes and signaling, amino acid metabolism, and carbohydrate metabolism (Supplementary Figure S2). Pairwise differences were observed for several PICRUST annotations: those belonging to metabolism of amino acids, biosynthesis of other secondary metabolites, carbohydrate metabolism, glycan biosynthesis and metabolism, metabolism of cofactors and vitamins, metabolism of other amino acids, and metabolism of terpenoids and polyketides, as well as for human cancer and metabolic diseases, decreased at T2 (Table 5).

## 4. Discussion

In the present study, a brief lifestyle intervention consisting of a plant-based diet, exercise, and stress management resulted in positive changes in markers of CV health and large changes in the gut microbial milieu after only six days. Furthermore, there were significant correlations between individual markers of cardiovascular health with bacterial taxa, suggesting changes in the microbiome are linked to changes in cardiovascular health. The intervention resulted in significant reductions in TC, TG, and LDL, as well as SBP and DBP with minimal changes in BMI. This suggests the mechanism of change in CV markers was independent of weight loss.

Compared to the standard American diet, plant-based diets are associated with lower risk of CVD, all-cause mortality, and decreases in individual markers of poor CV health [45,46,47]. However, few randomized controlled trials have tested the effects of plant-based diets on changes in markers of CV health and gut microbiota composition and functional activity in individuals not following plant-based diets. Such studies are needed to tease out the potential role of other lifestyle factors in contributing to health among individuals following a plant-based diet.

Within the gut microbiota, there was an abundance of Firmicutes and Bacteroidetes in most adults at baseline, as expected on the standard American diet. Samples collected at T2 after the plant-based dietary intervention were largely characterized by increased abundances of members of Clostridia, a commensal class of Firmicutes consisting of gram-positive bacteria crucial to gut homeostasis [48]. We also detected favorable changes associated with improved metabolic profiles, notably surrounding the production of short chain fatty acids (SCFAs), including butyrate. Noteworthy, butyrate has been linked with improved cardio-metabolic health and remains of particular interest due to its anti-inflammatory properties. The physiological functions of butyrate are multifold and include provision of energy to colonocytes as well as fortification of the gut barrier via upregulation of tight junctions between colonocytes. The effects of butyrate on the gut barrier lead to decreased translocation of lipopolysaccharide (LPS) into systemic circulation and thus decreases in systemic inflammation [49]. In addition to localized effects in the gut, Wang et al. showed that butyrate decreased the expression of both TNF-a induced cytokines and vascular endothelial monocyte adhesion molecules in vitro, suggesting a potential mechanism by which butyrate mitigates cardiovascular disease [50]. The decreases we observed in *Bacteroides* appear reflective of the increased diversification of the gut flora as well as a favorable shift towards *Ruminococcaceae*. With the intervention, there was also an increase in *Lachnospiraceae* (median increase of 56.7%) and *Ruminococcaceae* (median increase of 131%), both of which are families that comprise well known butyrate producing bacteria, in patients with high risk of heart disease after just six days. Many species of *Lachnospiraceae* produce SCFAs, including acetate, butyrate, and propionate, through their fermentation of dietary fiber [51]. It was recently discovered that a deficiency in *Lachnospiraceae NK4B4* and *UCG-004* characterizes the fecal microbiome of patients with advanced coronary artery disease, after adjusting for diabetes and dyslipidemia, compared to healthy controls [52].

Both *F. prausnitzii* and *Faecalibacterium CCM04-06* increased following the dietary intervention. *F. prausnitzii*, a member of the *Ruminococcaceae* family, is of high interest in many studies surrounding gut microbiota due to its anti-inflammatory properties, and was recently shown to exert its anti-inflammatory effects through production of butyrate [53]. In an 8-week, parallel-group, randomized control trial of overweight and obese individuals who adopted a Mediterranean diet, Meslier et al. observed decreases in plasma cholesterol and increases in *F. prausnitzii* as well as increases in the associated bacterial genes implicated in carbohydrate degradation and increased butyrate production [54]. The decreases in plasma LDL were proportional to adherence to the study diet. Although our study did not detect a significant negative correlation between *F. prausnitzii* and blood pressure, such a correlation has been demonstrated previously in a nutritional intervention study with a longer duration of intervention [55].

In this study, we observed a 9.43 log2fold change higher abundance in the mucus colonizer *Akkermansia muciniphila (A. muciniphila)* in individuals with low serum hsCRP, indicative of nearly 700% higher colonization in low hsCRP subjects compared to those with high hsCRP. *A. muciniphila* has anti-inflammatory properties and its abundance has been negatively correlated with obesity, diabetes, and metabolic syndromes in human studies. The mechanisms of interaction of this bacterium with the human host are not clear; however, there is growing evidence of its health-promoting capabilities. In vitro studies have demonstrated that *A. muciniphila* adhesion to the intestinal epithelium improves enterocyte monolayer integrity [56]. Patients with T2DM have lower *A. muciniphila* abundance and gut barrier permeability dysfunction [57]. After weight loss, type 2 diabetics have shown an increase in *F. prausnitzii*, and *A. muciniphila* [58].

In our study, we found that TMAO measurement inversely correlated with relative abundances of *Bifidobacterium*, *Alistipes*, *Eubacterium*, *Ruminiclostridium 9*, and *Dialister* both before and after the intervention. *Rothia*, a gluten-consuming bacterium of the family *Micrococcaceae* that naturally colonizes the upper gastrointestinal tract [59], *Veillonella*, and *Ruminococcaceae UCG-003* abundances were positively correlated with TMAO measurements at both timepoints. Some species of *Clostridia* and *Proteobacteria* are known to consume choline, and have been implicated in the generation and accumulation of TMA; at least eight species of *Firmicutes* and *Proteobacteria* have been shown to produce TMA from choline in vitro [60]. While we did not detect significant differences in plasma TMAO concentrations within the intervention period, the correlation between the microbiota changes and TMAO is notable. The absence of significant change in TMAO from this plant-based intervention is consistent with the literature suggesting that the role in TMAO in cardiovascular health is unclear.

### Limitations

Although there are many strengths (replication by an independent cohort, significant changes in many markers of CV risk in only six-days, etc.), there are some limitations worthy of mention. Lack of a randomized comparator group eliminates the possibility to assess causation but provides important preliminary data for hypothesis generation and future study design. An intervention period of only six-days is likely too brief to appreciate the full physiological effects, and the lack of longer follow-up precludes the ability to determine if changes observed are lasting. While 16s rRNA sequencing examines a single gene in every bacterium, whole metagenome sequencing examines the entire genomic content of the gut microbiota in an unbiased approach. This approach would have provided accurate species level identity of the microbes, as well as their complete genome. Such analysis can reveal the metabolic potential of these communities, providing a more complete picture of their function as a whole [61]. Studies of diet-induced gut microbiota changes are ongoing.

## 5. Conclusions

Lifestyle-based interventions lasting as brief as one week can produce significant improvements in lipids and BP without weight loss and are associated with changes in intestinal microbiota. Without weight loss as a potential mechanism, we must consider that these clinical improvements may be due to complex changes in microbiota composition. Further study is needed to assess the impact of these changes on other parameters of cardiovascular health and adherence to dietary change over longer periods of time. Future analyses of this work involving dietary intake, physical activity, and quality of life measures at later time points may reveal sustainable effects of this short intervention. Microbiota changes were related to reductions in cardiometabolic risk factors, which supports the need for further study of dietary and oral therapies that can similarly alter microbiota composition to add in improvement of CV health.

## Figures and Tables

**Table 1:** Demographics and difference in clinical variables over time. Systolic blood pressure, SBP; diastolic blood pressure, DBP; total cholesterol, T; triglycerides, TRG; high density lipoprotein, HDL; low density lipoprotein, LDL; very low density lipoprotein, VLDL; high-sensitivity C-reactive protein, hsCRP; trimethylamine N-oxide, TMAO; body mass index, BMI; standard deviation, Std; Benjamini-Hochberg critical value, BH.

	Baseline ^1^		Day 6 ^1^		Difference			
	Mean	Std	Mean	Std	Mean	Std	*p*	BH
Age (years)	46.89	12.38	-	-	-	-	-	-
BMI	31.14	8.83	31.21	9.75	−0.47	0.39	<0.0001	0.0125
Height (cm)	169.19	8.13	-	-	-	-	-	-
Weight (kg)	89.59	27.58	90.24	31.86	−1.35	1.15	<0.0001	0.025
Waist (cm)	104.90	23.32	102.21	21.06	−2.69	10.95	0.0408	0.1500
Hip (cm)	114.33	19.56	113.23	18.54	−1.09	4.37	0.0372	0.1375
SBP (mmHg)	129.92	17.52	125.46	14.82	−4.46	15.63	0.0181	0.1250
DBP (mmHg)	84.76	10.36	81.39	8.27	−3.38	10.17	0.0063	0.1125
TC (mg/dL)	184.15	33.36	167.26	32.86	−16.89	16.39	<0.0001	0.0375
TRG (mg/dL)	132.18	76.12	107.38	51.89	−24.81	46.04	<0.0001	0.0750
HDL (mg/dL)	52.90	15.25	51.07	14.75	−1.83	4.98	0.0026	0.1000
LDL (mg/dL)	104.81	28.48	94.99	27.53	−9.82	15.61	<0.0001	0.0500
VLDL (mg/dL)	26.46	15.21	21.47	10.38	−4.99	9.17	<0.0001	0.0625
LDL/HDL	2.16	0.91	2.02	0.87	−0.14	0.35	0.0016	0.0875
hsCRP	2.32	2.16	2.51	2.82	−0.23	1.66	0.2905	0.1625
Glucose	99.81	31.94	97.90	23.87	−1.90	16.09	0.3191	0.1750
TMAO	4.58	8.12	4.73	8.34	0.14	3.39	0.7488	0.1875

^1^ *N* = 71 for BMI and weight. *N* = 61 for hsCRP, *N* = 65 for TMAO. *N* = 72 for all others.

**Table 2:** Baseline characteristics of hypertensive versus normotensive participants at day 0. Body mass index, BMI; Systolic blood pressure, SBP; diastolic blood pressure, DBP; high density lipoprotein, HDL; low density lipoprotein, LDL; very low density lipoprotein, VLDL; trimethylamine N-oxide, TMAO.

	Hypertensive (SBP ≥ 130/80 mmHg)	<130/80 mmHg
No. of participants (% of total)	56 (76.7%)	17 (23.3%)
Average age (years)	48.41	41.75
Male (% of total)	22 (39.3%)	15 (88.2%)
Female (% of total)	34 (60.7%)	2 (11.8%)
No. Taking antihypertensive medications (% of total)	17 (21.25%)	2 (2.5%)
Mean BMI (kg/m^2^)	31.96	28.33
Mean height (cm)	170.51	164.49
Mean weight (kg)	93.35	76.65
Mean waist size (cm)	108.43	92.51
Mean hip size (cm)	116.33	107.24
Mean waist to hip ratio	0.93	0.86
Mean total cholesterol (mg/dL)	184	185
Mean triglycerides (mg/dL)	131	138
Mean HDL (mg/dL)	51	59
Mean LDL (mg/dL)	107	98
Mean LDL/HDL Ratio	2.25	1.83
Mean VLDL (mg/dL)	26	28
High sensitivity C-reactive protein	3.74	4.39
Glucose	103	87
TMAO	4.03	6.16

**Table 3:** Microbiome shifts within individual. Pairwise Wilcoxon statistics for MT cohort (N = 22). The 20 most abundant taxa (23 families, 23 genera, 27 species, and 24 ASVs) at each level were compared. Median relative abundance (as a percentage of the microbiome composition) and percent change of the families, genera, species, and ASVs that differed significantly within-person across the two time points are presented for the taxonomic differences with a *p* value < 0.1. Taxa that increased or decreased between timepoints are indicated in green and red, respectively. Statistics for all pairwise tests are provided in Supplementary Table S1. Taxonomic assignments are based on the Silva 138 database. The sequences for the ASVs that were tested are provided in Supplementary Material (Supplementary Table S2). ASV, amplicon sequence variant.

Phylum	Class	Order	Family	Genus	Species	ASV	T1	T2	%Diff	*p*
**Family-level**										
Firmicutes	Clostridia	Lachnospirales	*Lachnospiraceae*				17.53%	27.83%	58.78%	0.0002
Firmicutes	Clostridia	Oscillospirales	*Ruminococcaceae*				9.32%	16.97%	82.05%	0.0003
Firmicutes	Clostridia	Monoglobales	*Monoglobaceae*				0.53%	1.15%	117.39%	0.0004
Actinobacteriota	Coriobacteriia	Coriobacteriales	*Eggerthellaceae*				0.74%	1.28%	73.96%	0.002
Firmicutes	Clostridia	Christensenellales	*Christensenellaceae*				0.27%	0.70%	157.75%	0.001
Firmicutes	Clostridia	Oscillospirales	*Butyricicoccaceae*				0.39%	0.75%	92.08%	0.023
Firmicutes	Bacilli	Erysipelotrichales	*Erysipelatoclostridiaceae*				0.15%	0.38%	157.89%	0.054
Proteobacteria	Gammaproteobacteria	Enterobacterales	*Enterobacteriaceae*				0.22%	0.37%	66.67%	0.095
Bacteroidota	Bacteroidia	Bacteroidales	*Barnesiellaceae*				0.16%	0.01%	−92.86%	0.001
Proteobacteria	Gammaproteobacteria	Burkholderiales	*Sutterellaceae*				0.36%	0.21%	−42.55%	0.0003
Bacteroidota	Bacteroidia	Bacteroidales	*Marinifilaceae*				0.21%	0.04%	−79.63%	0.0001
Desulfobacterota	Desulfovibrionia	Desulfovibrionales	*Desulfovibrionaceae*				0.21%	0.05%	−78.18%	0.001
Bacteroidota	Bacteroidia	Bacteroidales	*Tannerellaceae*				0.97%	0.43%	−54.98%	0.001
Bacteroidota	Bacteroidia	Bacteroidales	*Rikenellaceae*				1.24%	0.58%	−53.56%	0.013
Firmicutes	Negativicutes	Acidaminococcales	*Acidaminococcaceae*				13.99%	3.12%	−77.74%	0.025
Bacteroidota	Bacteroidia	Bacteroidales	*Bacteroidaceae*				27.58%	11.83%	−57.09%	0.005
**Genus-level**										
Firmicutes	Clostridia	Oscillospirales	*Ruminococcaceae*	*Faecalibacterium*			6.57%	10.96%	66.86%	0.001
Firmicutes	Clostridia	Lachnospirales	*Lachnospiraceae*	*Roseburia*			0.50%	2.06%	308.40%	0.002
Firmicutes	Clostridia	Lachnospirales	*Lachnospiraceae*	*Lachnospiraceae NK4A136 group*			0.33%	1.70%	418.82%	0.0004
Firmicutes	Clostridia	Oscillospirales	*Ruminococcaceae*	*Subdoligranulum*			0.60%	1.91%	220.65%	0.001
Firmicutes	Clostridia	Lachnospirales	*Lachnospiraceae*	*Anaerostipes*			0.63%	1.94%	205.45%	0.007
Firmicutes	Clostridia	Lachnospirales	*Lachnospiraceae*	*Blautia*			2.18%	3.30%	51.06%	0.011
Firmicutes	Clostridia	Oscillospirales	*Ruminococcaceae*	*Ruminococcus*			0.80%	1.85%	130.14%	0.028
Firmicutes	Clostridia	Monoglobales	*Monoglobaceae*	*Monoglobus*			0.53%	1.15%	117.39%	0.0004
Firmicutes	Negativicutes	Veillonellales-Selenomonadales	*Veillonellaceae*	*Veillonella*			0.20%	0.76%	271.70%	0.018
Firmicutes	Clostridia	Christensenellales	*Christensenellaceae*	*Christensenellaceae R-7 group*			0.23%	0.69%	198.33%	0.001
Firmicutes	Clostridia	Oscillospirales	*Butyricicoccaceae*	*Butyricicoccus*			0.39%	0.75%	92.08%	0.020
Actinobacteriota	Coriobacteriia	Coriobacteriales	*Eggerthellaceae*	*Adlercreutzia*			0.40%	0.65%	60.00%	0.004
Bacteroidota	Bacteroidia	Bacteroidales	*Tannerellaceae*	*Parabacteroides*			0.97%	0.43%	−54.98%	0.001
Bacteroidota	Bacteroidia	Bacteroidales	*Rikenellaceae*	*Alistipes*			1.24%	0.53%	−57.59%	0.013
Firmicutes	Negativicutes	Acidaminococcales	*Acidaminococcaceae*	*Phascolarctobacterium*			13.99%	3.12%	−77.74%	0.029
Bacteroidota	Bacteroidia	Bacteroidales	*Bacteroidaceae*	*Bacteroides*			27.58%	11.83%	−57.09%	0.005
**Species-level**										
Firmicutes	Clostridia	Oscillospirales	*Ruminococcaceae*	*Faecalibacterium*	*Faecalibacterium prausnitzii*		4.83%	7.47%	54.49%	0.002
Firmicutes	Clostridia	Lachnospirales	*Lachnospiraceae*	*Blautia*	*Blautia obeum*		0.33%	0.80%	141.86%	0.004
Firmicutes	Clostridia	Lachnospirales	*Lachnospiraceae*	*Anaerostipes*	*Anaerostipes hadrus*		0.35%	0.68%	93.48%	0.013
Firmicutes	Clostridia	Lachnospirales	*Lachnospiraceae*	*Lachnospiraceae NK4A136 group*	*Lachnospiraceae NK4A136 group bacterium*		0.10%	0.35%	260.00%	0.003
Actinobacteriota	Coriobacteriia	Coriobacteriales	*Eggerthellaceae*	*Adlercreutzia*	*Adlercreutzia equolifaciens*		0.11%	0.29%	167.86%	0.020
Firmicutes	Clostridia	Lachnospirales	*Lachnospiraceae*	*Roseburia*	*Roseburia hominis*		0.07%	0.22%	222.22%	0.0002
Firmicutes	Clostridia	Lachnospirales	*Lachnospiraceae*	*Blautia*	*Blautia faecis*		0.21%	0.35%	63.64%	0.0004
Firmicutes	Negativicutes	Veillonellales-Selenomonadales	*Veillonellaceae*	*Veillonella*	*Veillonella dispar*		0.06%	0.20%	218.75%	0.033
Bacteroidota	Bacteroidia	Bacteroidales	*Bacteroidaceae*	*Bacteroides*	*Bacteroides thetaiotaomicron*		0.35%	0.46%	30.77%	0.038
Firmicutes	Clostridia	Lachnospirales	*Lachnospiraceae*	*Dorea*	*Dorea formicigenerans*		0.13%	0.19%	40.00%	0.064
Firmicutes	Clostridia	Lachnospirales	*Lachnospiraceae*	*Coprococcus*	*Coprococcus comes*		0.23%	0.13%	−40.68%	0.044
Desulfobacterota	Desulfovibrionia	Desulfovibrionales	*Desulfovibrionaceae*	*Bilophila*	*Bilophila wadsworthia*		0.17%	0.03%	−81.40%	0.002
Bacteroidota	Bacteroidia	Bacteroidales	*Bacteroidaceae*	*Bacteroides*	*Bacteroides caccae*		0.32%	0.17%	−47.56%	0.004
Bacteroidota	Bacteroidia	Bacteroidales	*Tannerellaceae*	*Parabacteroides*	*Parabacteroides merdae*		0.20%	0.01%	−96.08%	0.036
Bacteroidota	Bacteroidia	Bacteroidales	*Tannerellaceae*	*Parabacteroides*	*Parabacteroides distasonis*		0.37%	0.12%	−67.37%	0.002
Firmicutes	Clostridia	Lachnospirales	*Lachnospiraceae*	*Blautia*	*Blautia massiliensis*		0.50%	0.22%	−56.15%	0.099
Bacteroidota	Bacteroidia	Bacteroidales	*Rikenellaceae*	*Alistipes*	*Alistipes putredinis*		0.73%	0.15%	−79.47%	0.001
Bacteroidota	Bacteroidia	Bacteroidales	*Bacteroidaceae*	*Bacteroides*	*Bacteroides vulgatus*		5.71%	2.71%	−52.49%	0.003
**ASV-level**										
Firmicutes	Clostridia	Oscillospirales	*Ruminococcaceae*	*Faecalibacterium*	*Faecalibacterium prausnitzii*	13	0.50%	1.49%	195.42%	0.001
Firmicutes	Clostridia	Monoglobales	*Monoglobaceae*	*Monoglobus*		16	0.37%	1.08%	195.79%	0.0004
Firmicutes	Clostridia	Lachnospirales	*Lachnospiraceae*	*Blautia*	*Blautia obeum*	25	0.33%	0.80%	141.86%	0.004
Firmicutes	Clostridia	Oscillospirales	*Ruminococcaceae*	*Faecalibacterium*		15	0.57%	1.00%	76.19%	0.033
Firmicutes	Clostridia	Lachnospirales	*Lachnospiraceae*			20	0.48%	0.86%	76.98%	0.0004
Firmicutes	Clostridia	Lachnospirales	*Lachnospiraceae*	*Anaerostipes*	*Anaerostipes hadrus*	32	0.21%	0.53%	157.41%	0.017
Firmicutes	Clostridia	Lachnospirales	*Lachnospiraceae*			37	0.24%	0.52%	114.29%	0.004
Firmicutes	Clostridia	Oscillospirales	*Ruminococcaceae*	*Faecalibacterium*	*Faecalibacterium prausnitzii*	2	1.57%	1.77%	12.50%	0.067
Bacteroidota	Bacteroidia	Bacteroidales	*Bacteroidaceae*	*Bacteroides*	*Bacteroides thetaiotaomicron*	22	0.29%	0.46%	58.67%	0.046
Firmicutes	Clostridia	Oscillospirales	*Ruminococcaceae*	*Subdoligranulum*		24	0.33%	0.50%	49.43%	0.027
Firmicutes	Clostridia	Oscillospirales	*Ruminococcaceae*	*Subdoligranulum*		43	0.19%	0.35%	82.00%	0.052
Firmicutes	Clostridia	Lachnospirales	*Lachnospiraceae*	*Agathobacter*		5	2.07%	2.11%	2.04%	0.059
Firmicutes	Clostridia	Lachnospirales	*Lachnospiraceae*	*Blautia*	*Blautia massiliensis*	27	0.50%	0.22%	−56.15%	0.099
Bacteroidota	Bacteroidia	Bacteroidales	*Bacteroidaceae*	*Bacteroides*	*Bacteroides uniformis*	7	1.06%	0.61%	−42.75%	0.036
Bacteroidota	Bacteroidia	Bacteroidales	*Rikenellaceae*	*Alistipes*	*Alistipes putredinis*	40	0.73%	0.15%	−79.47%	0.001
Bacteroidota	Bacteroidia	Bacteroidales	*Bacteroidaceae*	*Bacteroides*	*Bacteroides vulgatus*	8	1.28%	0.68%	−46.69%	0.005
Bacteroidota	Bacteroidia	Bacteroidales	*Bacteroidaceae*	*Bacteroides*	*Bacteroides vulgatus*	3	2.97%	0.73%	−75.42%	0.019

**Table 4:** Differentially abundant genera across clinical subgroups at T2. Taxa with differential abundance across hypertension, LDL, or hsCRP subgroups at T2, based on DESeq2 analysis. The subgroup with higher relative abundance is indicated for each clinical comparison, with log2fold change (log2FC), log fold change standard error (lfcSE), *p* value, and *p* value after false discovery rate correction (FDR). ASV1 is mapped to Firmicutes | Negativicutes|Acidaminococcales | *Acidaminococcaceae* | *Phascolarctobacterium* | *Phascolarctobacterium faecium*. ASV837 is mapped to Firmicutes|Bacilli|Lactobacillales | *Lactobacillaceae* | *Lactobacillus* | *Lactobacillus paracasei*. Low density lipoprotein, LDL; high-sensitivity C-reactive protein, hsCRP; blood pressure, BP.

Taxon	Taxonomic Level	Measure	Higher Abundance	log2FC	lfcSE	*p*	FDR
*Akkermansia*	Genus	LDL	Desirable	−8.84	2.30	<0.001	0.005
*Akkermansia muciniphila*	Species	hsCRP	Low	9.43	2.32	<0.001	0.004
Akkermansiaceae	Family	LDL	Desirable	−8.91	2.25	<0.001	0.002
*Alistipes onderdonkii*	Species	hsCRP	High	−4.65	1.60	0.004	0.130
*Alistipes onderdonkii*	Species	LDL	High	5.61	1.79	0.002	0.029
ASV1	ASV	LDL	Desirable	−8.31	1.45	<0.001	<0.001
ASV837	ASV	hsCRP	Low	7.05	2.11	<0.001	0.038
*Lactobacillaceae*	Family	hsCRP	Low	4.82	1.38	<0.001	0.029
*Lactobacillus*	Genus	hsCRP	Low	4.76	1.42	0.001	0.129
*Lactobacillus paracasei*	Species	hsCRP	Low	7.00	1.80	<0.001	0.006
*Phascolarctobacterium*	Genus	LDL	Desirable	−7.37	1.21	<0.001	<0.001
*Phascolarctobacterium faecium*	Species	LDL	Desirable	−8.28	1.24	<0.001	<0.001
*Prevotella copri*	Species	LDL	High	9.56	3.52	0.007	0.093
*Prevotella undetermined*	Species	LDL	High	10.22	3.18	0.001	0.025
*Romboutsia*	Genus	BP	Hypertensive or elevated	−3.28	1.26	0.009	0.117
*Romboutsia ilealis*	Species	BP	Hypertensive or elevated	−3.43	1.31	0.009	0.110

**Table 5:** PICRUSt functional changes within individual across the intervention. Wilcoxon paired test results for PICRUSt functional annotations across T1 and T2. Phylogenetic Investigation of Communities by Reconstruction of Unobserved States, PICRUSt.

PICRUSt Annotation	T1 Median	T2 Median	*z*	*p*
Human Diseases; Cancers	0.00097	0.00092	−3.62	0.0003
Human Diseases; Metabolic Diseases	0.00106	0.00099	−4.01	<0.00001
Metabolism; Amino Acid Metabolism	0.10014	0.09853	3.43	0.0006
Metabolism; Biosynthesis of Other Secondary Metabolites	0.01037	0.00961	−2.74	0.0061
Metabolism; Carbohydrate Metabolism	0.11120	0.10954	−2.32	0.0203
Metabolism; Glycan Biosynthesis and Metabolism	0.02372	0.01953	−3.75	0.0002
Metabolism; Metabolism of Cofactors and Vitamins	0.04409	0.04324	−3.36	0.0008
Metabolism; Metabolism of Other Amino Acids	0.01515	0.01466	−3.17	0.0015
Metabolism; Metabolism of Terpenoids and Polyketides	0.01645	0.01612	−3.39	0.0007

## Data Availability

The anonymized data presented in this study are openly available in NCBI BioProject (accession number PRJNA766548) at https://www.ncbi.nlm.nih.gov/bioproject/PRJNA766548.

## Supplementary Materials

The following are available online at https://www.mdpi.com/article/10.3390/nu13103459/s1, Text 1: Detailed Immersion program menu. Figure S1: Distinct microbial communities present at 40% prevalence in low hsCRP and high hsCRP groups at T2, Figure S2: PICRUSt functional profiles across time, Table S1: Wilcoxon statistics for all pairwise tests across taxonomic levels, Table S2: Taxonomic assignment (based on the Silva 138 database) and full sequences for those amplicon sequence variants (ASVs) that contributed significantly to the shifts in the gut microbiome across the two time points, Table S3: Amplicon sequence variants contributing to random forest model in PIME, Table S4: Amplicon sequence variants contributing to the classification of hsCRP subgroup at T2 at 40% prevalence.
