# Supplementary material for: A Six-Day, Lifestyle-Based Immersion Program Mitigates Cardiovascular Risk Factors and Induces Shifts in Gut Microbiota, Specifically Lachnospiraceae, Ruminococcaceae, Faecalibacterium prausnitzii: A Pilot Study
Source: Nutrients. 2021 Sep 29;13(10):3459. doi: 10.3390/nu13103459 (PMC8539164; doi:10.3390/nu13103459)

SUPPLEMENTARY MATERIAL

Text 1. Detailed Immersion program menu

Day 1 – reception

- Crudité of fresh vegetables, whole grain crostini, toasted whole wheat pita
- Assortment of dips: cannellini beans, fava beans, hummus, spinach and roasted red pepper, cauliflower
- Assorted fruit and vegetable infused water: lemon, lime, cucumber and mint

Day 1 – dinner (American theme)

- Salad bar
  - romaine lettuce, baby Florida greens, kale, spinach, arugula (rotate two greens per salad bar) sliced tomatoes, kidney beans, sliced radishes, sliced cucumbers, shredded carrots, shredded red cabbage, sliced onions, diced avocado, chick peas, snow peas, tri-colored peppers, chopped zucchini, corn, radicchio, dill, parsley
  - lightly sautéed mushrooms in vegetable stock
  - spices: nutritional yeast, red pepper flakes, dulse/kelp, cayenne pepper, garlic powder, black pepper
  - nuts and seeds (walnuts, almonds, pecans, Brazil nuts, cashews, pistachios and flax, chia, sesame, sunflower, and pumpkin; rotate two nuts and two seeds each day)
  - dressings: three flavored vinegars, house-made cashew Cesar salad dressing
- Soup: red bean and turnip chili
- Entrée
  - veggie burger in whole wheat pita pockets
  - toppings: sliced tomato, avocado, onions
  - condiments: mustard, hummus (without oil) and organic ketchup, sugar-free Dijon mustard
- Side dishes (greens)
  - steamed mixed veggies (asparagus, squash, carrots)
  - pineapple spinach salad
- Side dish (starch)
  - sweet potatoes gratin topped with almond butter
- Dessert
  - chocolate cake
  - cut fruit: pineapple, watermelon, cantaloupe, honeydew, and berries
  - whole fruits: apples, oranges, bananas, pears, and plums
- Beverages
  - water and herbal tea with lemon wedges and unsweetened plant-based milks

Day 2 – breakfast

- Grain
  - steel cut plain oatmeal (hot cereal)
  - blueberry oatmeal (cold cereal)

- Smoothie: spinach, bananas, and strawberries
- Fruit and berries
  - berries (blueberries, strawberries, raspberries, blackberries; rotate two of 4=four each day)
  - cut fruit: pineapple, watermelon, cantaloupe, and honeydew
  - whole fruit: apples, oranges, bananas, pears, and plums
- Entrée: warm tropical quinoa
- Greens: romaine lettuce and quartered avocado (with skin intact)
- Nuts and seeds (walnuts, almonds, pecans, Brazil nuts, cashews, pistachios, flax, chia, sesame, sunflower, and pumpkin; rotate two nuts and two seeds each day)
- Spices: cinnamon, pumpkin, cayenne pepper, lemon wedges
- Beverages: water and herbal tea with lemon wedges and unsweetened plant-based milks

Day 2 – lunch

- Salad bar
  - romaine lettuce leaves, baby Florida greens, kale, spinach, arugula (rotate two greens per salad bar) sliced tomatoes, kidney beans, sliced radishes, sliced cucumbers, shredded carrots, shredded red cabbage, very thinly sliced onions, diced avocado, chick peas, snow peas, tri colored pepper, chopped zucchini, corn, radicchio, dill, parsley
  - lightly sautéed mushrooms in vegetable stock
- Spices: nutritional yeast, red pepper flakes, dulse/kelp, cayenne pepper, garlic powder, black pepper
- Nuts and seeds (walnuts, almonds, pecans, Brazil nuts, cashews, pistachios and flax, chia, sesame, sunflower, pumpkin; rotate two nuts and two seeds each day)
- Dressings
  - three flavored vinegars
  - house-made cashew Cesar salad dressing
- Soup: roasted red pepper soup
- Entrée
  - whole wheat veggie pesto wrap with eggplant, squashes, mushroom, and bean sprouts
  - whole wheat seaweed slaw wrap with tofu, avocados, roasted red pepper, and bean dip
- Side green dish
  - kale and avocado salad
- Side starch dish: three beans salad with pumpkin seeds
- Dessert
  - cut fruit (pineapple, watermelon, cantaloupe, honeydew, berries)
  - bowl of hand fruit (apples, oranges, bananas, pears, plums)
- Beverages
  - water and herbal tea w/ lemon wedges and unsweetened plant-based milks

Day 2 – dinner (Asian theme)

- Salad bar
  - romaine lettuce leaves, baby Florida greens, kale, spinach, arugula (rotate two greens per salad bar) sliced tomatoes, kidney beans, sliced radishes, sliced cucumbers, shredded carrots, shredded red cabbage, very

thinly sliced onions, diced avocado, chick peas, snow peas, tri colored pepper, chopped zucchini, corn, radicchio, dill, parsley

- o lightly sautéed mushrooms in vegetable stock

• Spices: nutritional yeast, red pepper flakes, dulse/kelp, cayenne pepper, garlic powder, black pepper

• Nuts and seeds (walnuts, almonds, pecans, Brazil nuts, cashews, pistachios and flax, chia, sesame, sunflower, pumpkin; rotate two nuts and two seeds each day)

• Dressings

- o three flavored vinegars
- o house-made cashew Cesar salad dressing

• Soup: shitake mushroom with hatasui (Chinese cabbage) broth

• Entrée: tahini rubbed tofu with baby bock choy and kabu (turnip) in ginger stock

• Side green dish

- o horenso (spinach), red cabbage renkon (lotus root), with lemon grass
- o steamed broccoli and cauliflower

• Side starch dish: red rice

• Dessert

- o cut fruit (pineapple, watermelon, cantaloupe, honeydew, berries)
- o bowl of hand fruit (apples, oranges, bananas, pears, plums)

• Beverages

- o water and herbal tea with lemon wedges and unsweetened plant-based milks

Day 3 – breakfast

• Grain

- o steel cut pineapple oatmeal (hot cereal)
- o muesli (cold cereal)

• Smoothie: peach and spinach

• Fruit and berries

- o berries (blueberries, strawberries, raspberries, and blackberries; rotate two of four each day)
- o cut fruit (pineapple, watermelon, cantaloupe, honeydew)
- o bowl of hand fruit (apples, oranges, bananas, pears, plums)

• Entrée: pancakes served with applesauce

• Greens: romaine leaves and quartered avocado (with skin intact)

• Nuts and seeds (walnuts, almonds, pecans, Brazil nuts, cashews, pistachios and flax, chia, sesame, sunflower, pumpkin; rotate two nuts and two seeds each day)

• Spices: cinnamon, pumpkin spice, cayenne pepper, lemon wedges

• Beverages: water and herbal tea with lemon wedges and unsweetened plant-based milks

Day 3 – lunch

• Salad bar

- o romaine lettuce leaves, baby Florida greens, kale, spinach, arugula (rotate two greens per salad bar) sliced tomatoes, kidney beans, sliced radishes, sliced cucumbers, shredded carrots, shredded

red cabbage, very thinly sliced onions, diced avocado, chick peas, snow peas, tri colored pepper, chopped zucchini, corn, radicchio, dill, parsley

- o lightly sautéed mushrooms in vegetable stock
  - Spices: nutritional yeast, red pepper flakes, dulse/kelp, cayenne pepper, garlic powder, black pepper
  - Nuts and seeds (walnuts, almonds, pecans, Brazil nuts, cashews, pistachios and flax, chia, sesame, sunflower, pumpkin; rotate two nuts and two seeds each day)
  - Dressings
- o three flavored vinegars
- o house-made cashew Cesar salad dressing
  - Soup: cream of mushroom soup
  - Entrée
- o sweet potato pie with roasted vegetables, mix baby greens topped with roasted mix nuts
  - Side green dish
- o cauliflower, kale and collard greens in coconut milk puree with giant Peruvian lima beans
  - Side starch dish: red quinoa with lentils and fresh cilantro
  - Dessert
- o cut fruit (pineapple, watermelon, cantaloupe, honeydew, berries)
- o whole fruit (apples, oranges, bananas, pears, plums)
  - Beverages
- o water and herbal tea with lemon wedges and unsweetened plant-based milks

Day 3 – dinner (Moroccan theme)

- Salad bar
- o romaine lettuce leaves, baby Florida greens, kale, spinach, arugula (rotate two greens per salad bar) sliced tomatoes, kidney beans, sliced radishes, sliced cucumbers, shredded carrots, shredded red cabbage, very thinly sliced onions, diced avocado, chick peas, snow peas, tri colored pepper, chopped zucchini, corn, radicchio, dill, parsley
- o lightly sautéed mushrooms in vegetable stock
  - Spices: nutritional yeast, red pepper flakes, dulse/kelp, cayenne pepper, garlic powder, black pepper
  - Nuts and seeds (walnuts, almonds, pecans, Brazil nuts, cashews, pistachios and flax, chia, sesame, sunflower, pumpkin; rotate two nuts and two seeds each day)
  - Dressings
- o three flavored vinegars
- o house-made cashew Cesar salad dressing
  - Soup: serrouda (Moroccan chickpea puree)
  - Entrée
- o tangine (vegetable stew) eggplant, carrots, peas, squashes, peppers, and tomato saffron sauce
- o toasted whole wheat pita points
  - Side green dish
- o sautéed fava beans and cauliflower in lemon zest, cumin and vegetable stock
- o steamed broccoli and cauliflower
  - Side starch dish: wheat couscous and lentils
  - Dessert
- o m'hancha cookies: almonds and dates with pastilla leave and water orange flowers
- o cut fruit (pineapple, watermelon, cantaloupe, honeydew, berries)

- bowl of hand fruit (apples, oranges, bananas, pears, plums)

• Beverages

- water and herbal tea with lemon wedges and unsweetened plant-based milks

Day 4 – breakfast

- Grain
  - steel cut plain oatmeal (hot cereal)
  - raisin oatmeal (cold cereal)
- Smoothie: cherry, chocolate, kale
- Fruit and berries
  - berries (blueberries, strawberries, raspberries, blackberries; rotate two each day)
  - cut fruit (pineapple, watermelon, cantaloupe, honeydew)
  - whole fruit (apples, oranges, bananas, pears, plums)
- Entrée: warm open face whole wheat pita with roasted squash, mushrooms, red peppers and hummus
- Greens: romaine leaves and quartered avocado (with skin intact)
- Nuts and seeds (walnuts, almonds, pecans, Brazil nuts, cashews, pistachios and flax, chia, sesame, sunflower, and pumpkin; rotate two nuts and two seeds each day)
- Spices: cinnamon, pumpkin, cayenne pepper, lemon wedges
- Beverages: water and herbal tea with lemon wedges and unsweetened plant-based milks

Day 4 – lunch

- Salad bar
  - romaine lettuce leaves, baby Florida greens, kale, spinach, arugula (rotate two greens per salad bar) sliced tomatoes, kidney beans, sliced radishes, sliced cucumbers, shredded carrots, shredded red cabbage, very thinly sliced onions, diced avocado, chick peas, snow peas, tri colored pepper, chopped zucchini, corn, radicchio, dill, parsley
  - lightly sautéed mushrooms in vegetable stock
- Spices: nutritional yeast, red pepper flakes, dulse/kelp, cayenne pepper, garlic powder, black pepper
- Nuts and seeds (walnuts, almonds, pecans, Brazil nuts, cashews, pistachios and flax, chia, sesame, sunflower, pumpkin; rotate two nuts and two seeds each day)
- Dressings
  - three flavored vinegars
  - house-made cashew Cesar salad dressing
- Soup: white bean and escarole
- Entrée: roasted vegetable balls in herbed tomato sauce (extra sauce)
- Side green dish: broccolini, mushrooms and cauliflower with casher butter sauce
- Side starch dish: tempeh and coarse polenta cakes
- Dessert
  - cut fruit (pineapple, watermelon, cantaloupe, honeydew, berries)
  - whole fruit (apples, oranges, bananas, pears, plums)
- Beverages
  - water and herbal tea with lemon wedges and unsweetened plant-based milks

Day 4 – dinner (Italian theme)

- Salad bar
  - romaine lettuce leaves, baby Florida greens, kale, spinach, arugula (rotate two greens per salad bar) sliced tomatoes, kidney beans, sliced radishes, sliced cucumbers, shredded carrots, shredded red cabbage, very thinly sliced onions, diced avocado, chick peas, snow peas, tri colored pepper, chopped zucchini, corn, radicchio, dill, parsley
  - lightly sautéed mushrooms in vegetable stock
- Spices: nutritional yeast, red pepper flakes, dulse/kelp, cayenne pepper, garlic powder, black pepper
- Nuts and seeds (walnuts, almonds, pecans, Brazil nuts, cashews, pistachios and flax, chia, sesame, sunflower, pumpkin; rotate two nuts and two seeds each day)
- Dressings
  - three flavored vinegars
  - house-made cashew Cesar salad dressing
- Soup: minestrone with buckwheat groats
- Entrée
  - veggie lasagna with red sauce (additional sauce on the side)
- Side green dish
  - summer Italian-style mixed vegetable salad
  - sautéed green cabbage with garlic, sun dried red pepper and cannellini beans
  - steamed broccoli and cauliflower
- Side starch dish: whole wheat penne pasta
- Dessert
  - cut fruit (pineapple, watermelon, cantaloupe, honeydew, berries)
  - whole fruit (apples, oranges, bananas, pears, plums)
- Beverages
  - water and herbal tea with lemon wedges and unsweetened plant-based milks

Day 5 – breakfast

- Grain
  - steel cut fig oatmeal (hot cereal)
  - strawberry oatmeal (cold cereal)
- Smoothie: banana, mango, kale
- Fruit and berries
  - berries (blueberries, strawberries, raspberries, blackberries; rotate two of four each day)
  - cut fruit (pineapple, watermelon, cantaloupe, honeydew)
  - whole fruit (apples, oranges, bananas, pears, plums)
- Entrée: sweet potato hash
- Greens: romaine leaves and quartered avocado (with skin intact)
- Nuts and seeds (walnuts, almonds, pecans, Brazil nuts, cashews, pistachios and flax, chia, sesame, sunflower, pumpkin; rotate two nuts and two seeds each day)
- Spices: cinnamon, pumpkin spice, cayenne pepper, lemon wedges
- Beverages: water and herbal tea with lemon wedges and unsweetened plant-based milks

Day 5 – lunch

- Salad bar
  - romaine lettuce leaves, baby Florida greens, kale, spinach, arugula (rotate two greens per salad bar) sliced tomatoes, kidney beans, sliced radishes, sliced cucumbers, shredded carrots, shredded red cabbage, very thinly sliced onions, diced avocado, chick peas, snow peas, tri colored pepper, chopped zucchini, corn, radicchio, dill, parsley
- lightly sautéed mushrooms in vegetable stock
- Spices: nutritional yeast, red pepper flakes, dulse/kelp, cayenne pepper, garlic powder, black pepper
- Nuts and seeds (walnuts, almonds, pecans, Brazil nuts, cashews, pistachios and flax, chia, sesame, sunflower, pumpkin; rotate two nuts and two seeds each day)
- Dressings
  - three flavored vinegars
  - house-made cashew Cesar salad dressing
- Soup: poblano corn chowder
- Entrée
  - 100% whole wheat pita-pizza (tofu, baby bock choy, marinara sauce)
  - 100% whole wheat pita-pizza (roasted vegetables, pesto sauce, black beans, corn, avocado, tomatillos sauce)
- Side green dish
  - kale, orange grapefruit, herbed vinaigrette, white beans, toasted almonds
- Side starch dish: green soba noodles salad with bamboo mushrooms, roasted red peppers tossed in sesame vinaigrette
- Dessert
  - cut fruit (pineapple, watermelon, cantaloupe, honeydew, berries)
  - whole fruit (apples, oranges, bananas, pears, plums)
- Beverages
  - water and herbal tea with lemon wedges and unsweetened plant-based milks

Day 5 – dinner (Caribbean theme)

- Salad bar
  - romaine lettuce leaves, baby Florida greens, kale, spinach, arugula (rotate two greens per salad bar) sliced tomatoes, kidney beans, sliced radishes, sliced cucumbers, shredded carrots, shredded red cabbage, very thinly sliced onions, diced avocado, chick peas, snow peas, tri colored pepper, chopped zucchini, corn, radicchio, dill, parsley
- Lightly sautéed mushrooms in vegetable stock
- Spices: nutritional yeast, red pepper flakes, dulse/kelp, cayenne pepper, garlic powder, black pepper
- Nuts and seeds (walnuts, almonds, pecans, Brazil nuts, cashews, pistachios and flax, chia, sesame, sunflower, pumpkin; rotate two nuts and two seeds each day)
- Dressings
  - three flavored vinegars
  - house-made cashew Cesar salad dressing
- Soup: four bean chili with tofu crumble
- Entrée
  - baked sole with fresh fennel and mango papaya salsa

- baked tofu with sauté mushrooms and herb gravy
  - Side green dish
- baked zucchini and squash with Provencal sauce
- broccoli and cauliflower
  - Side starch dish: quinoa roasted curry cauliflower and butternut squash
  - Dessert
- vanilla carrot/apple cake with macadamia nut icing
- cut fruit (pineapple, watermelon, cantaloupe, honeydew, berries)
- bowl of hand fruit (apples, oranges, bananas, pears, plums)
  - Beverages
- water and herbal tea with lemon wedges and unsweetened plant-based milks

Day 6 – breakfast

- Grain
  - steel cut oatmeal (hot cereal)
  - blackberry oatmeal (cold cereal)
- Smoothie: strawberries, blueberries, collard greens
- Fruit and berries
  - berries (blueberries, strawberries, raspberries, blackberries; rotate two of four each day)
  - cut fruit (pineapple, watermelon, cantaloupe, honeydew)
  - whole fruit (apples, oranges, bananas, pears, plums)
- Entrée: tofu scramble
- Greens: romaine leaves and quartered avocado (with skin intact)
- Nuts and seeds (walnuts, almonds, pecans, Brazil nuts, cashews, pistachios and flax, chia, sesame, sunflower, pumpkin; rotate two nuts and two seeds each day)
- Spices: cinnamon, pumpkin spice, cayenne pepper, lemon wedges
- Beverages: water and herbal tea with lemon wedges and unsweetened plant-based milks

Day 6 – lunch

- Salad bar
  - romaine lettuce leaves, baby Florida greens, kale, spinach, arugula (rotate two greens per salad bar) sliced tomatoes, kidney beans, sliced radishes, sliced cucumbers, shredded carrots, shredded red cabbage, very thinly sliced onions, diced avocado, chick peas, snow peas, tri colored pepper, chopped zucchini, corn, radicchio, dill, parsley
  - lightly sautéed mushrooms in vegetable stock
- Spices: nutritional yeast, red pepper flakes, dulse/kelp, cayenne pepper, garlic powder, black pepper
- Nuts and seeds (walnuts, almonds, pecans, Brazil nuts, cashews, pistachios and flax, chia, sesame, sunflower, pumpkin; rotate two nuts and two seeds each day)
- Dressings
  - three flavored vinegars
  - house-made cashew Cesar salad dressing
- Soup: garden vegetable soup
- Entrée: vegetable terrine (roasted vegetable brunoise with mixed beans and dried fruit relish
- side green dish
  - braised greens and kale with jicama
- Side starch dish: rosemary roasted purple potatoes
- Dessert
  - cut fruit (pineapple, watermelon, cantaloupe, honeydew, berries)
  - whole fruit (apples, oranges, bananas, pears, plums)
- Beverages
  - water and herbal tea with lemon wedges and unsweetened plant-based milks

Day 6 – dinner (Mexican theme)

- Salad bar
  - romaine lettuce leaves, baby Florida greens, kale, spinach, arugula (rotate two greens per salad bar) sliced tomatoes, kidney beans, sliced radishes, sliced cucumbers, shredded carrots, shredded red cabbage, very thinly sliced onions, diced avocado, chick peas, snow peas, tri colored pepper, chopped zucchini, corn, radicchio, dill, parsley
  - lightly sautéed mushrooms in vegetable stock
- Spices: nutritional yeast, red pepper flakes, dulse/kelp, cayenne pepper, garlic powder, black pepper
- Nuts and seeds (walnuts, almonds, pecans, Brazil nuts, cashews, pistachios and flax, chia, sesame, sunflower, pumpkin; rotate two nuts and two seeds each day)
- Dressings
  - three flavored vinegars
  - house-made cashew Cesar salad dressing
- Soup: black bean bisque
- Entrée: fajita station
  - roasted veggies: zucchini, squash, and mushrooms
  - red and green bell peppers and onion
  - salsa, guacamole, pinto beans, mango salsa, coconut cilantro cream
  - 100% whole wheat soft tortillas
- Side green dish
  - tomato, kale, corn salad
  - broccoli and cauliflower
- Side starch dish: Mexican brown rice
- Dessert
  - avocado chocolate pudding topped with berries and cashew cream
  - cut fruit (pineapple, watermelon, cantaloupe, honeydew, berries)
  - bowl of hand fruit (apples, oranges, bananas, pears, plums)
- Beverages
  - water and herbal tea with lemon wedges and unsweetened plant-based milks

Day 7 – breakfast

- Grain
  - steel cut plain oatmeal (hot cereal)
  - mixed berry oatmeal (cold cereal)
- Smoothie: kale, spinach, banana, blueberry
- Fruit and berries
  - berries (blueberries, strawberries, raspberries, blackberries; rotate two of four each day)
  - cut fruit (pineapple, watermelon, cantaloupe, honeydew)
  - whole fruit (apples, oranges, bananas, pears, plums)
- Entrée: tempeh hash
- Greens: romaine leaves and quartered avocado (with skin intact)
- Nuts and seeds (walnuts, almonds, pecans, Brazil nuts, cashews, pistachios and flax, chia, sesame, sunflower, pumpkin; rotate two nuts and two seeds each day)
- Spices: cinnamon, pumpkin spice, cayenne pepper, lemon wedges

• Beverages: water and herbal tea w/ lemon wedges and unsweetened plant-based milks

Day 7 – lunch

• Salad bar

- romaine lettuce leaves, baby Florida greens, kale, spinach, arugula (rotate two greens per salad bar) sliced tomatoes, kidney beans, sliced radishes, sliced cucumbers, shredded carrots, shredded red cabbage, very thinly sliced onions, diced avocado, chick peas, snow peas, tri colored pepper, chopped zucchini, corn, radicchio, dill, parsley
- lightly sautéed mushrooms in vegetable stock

• Spices: nutritional yeast, red pepper flakes, dulse/kelp, cayenne pepper, garlic powder, black pepper

• Nuts and seeds (walnuts, almonds, pecans, Brazil nuts, cashews, pistachios and flax, chia, sesame, sunflower, pumpkin; rotate two nuts and two seeds each day)

• Dressings

- three flavored vinegars
- house-made cashew Cesar salad dressing

• Soup: butternut squash

• Entrée: whole wheat/gluten free elbow pasta with almond chili sauce, shredded veggies, cilantro, basil and mint

• Side green dish: grilled asparagus, steamed French green beans and roasted cherry tomatoes

• Side starch dish: black turtle bean stew with wild rice

• Dessert

- cut fruit (pineapple, watermelon, cantaloupe, honeydew, berries)
- whole fruit (apples, oranges, bananas, pears, plums)

• Beverages

- water and herbal tea with lemon wedges and unsweetened plant-based milks

Supplementary Table S1. Microbiome shifts within individual. Pairwise Wilcoxon statistics for MT cohort (N = 22) for all tests conducted. The 20 most abundant taxa at each level were compared (23 families, 23 genera, 27 species, and 24 ASVs). Median relative abundance (as a percentage of the microbiome composition) and percent change of the families, genera, species, and ASVs that differed significantly within-person across the two time points are presented. Taxa that increased or decreased between timepoints are indicated in green and red, respectively, as in Table 3. All taxonomic assignments, including the ASVs, are based on the Silva 138 database. The ASV sequences that were tested are provided in Supplementary Material (Supplementary Table S2). ASV, amplicon sequence variant.

Phylum	Class	Order	Family	Genus	Species	ASV	T1	T2	% Diff	*z*	*p*
**Family-level**											
Firmicutes	Clostridia	Lachnospirales	*Lachnospiraceae*				17.53%	27.83%	10.30%	-3.68	0.0002
Firmicutes	Clostridia	Oscillospirales	*Ruminococcaceae*				9.32%	16.97%	7.65%	-3.65	0.0003
Firmicutes	Clostridia	Monoglobales	*Monoglobaceae*				0.53%	1.15%	0.62%	-3.53	0.0004
Actinobacteriota	Coriobacteriia	Coriobacteriales	*Eggerthellaceae*				0.74%	1.28%	0.55%	-3.07	0.002
Firmicutes	Negativicutes	Veillonellales-Selenomonadales	*Veillonellaceae*				0.79%	1.28%	0.49%	-0.08	0.936
Firmicutes	Clostridia	Christensenellales	*Christensenellaceae*				0.27%	0.70%	0.43%	-3.28	0.001
Firmicutes	Clostridia	Oscillospirales	*Butyricicoccaceae*				0.39%	0.75%	0.36%	-2.27	0.023
Actinobacteriota	Actinobacteria	Bifidobacteriales	*Bifidobacteriaceae*				0.47%	0.74%	0.27%	-0.63	0.529
Firmicutes	Bacilli	Erysipelotrichales	*Erysipelatoclostridiaceae*				0.15%	0.38%	0.23%	-1.93	0.054
Proteobacteria	Gammaproteobacteria	Enterobacterales	*Enterobacteriaceae*				0.22%	0.37%	0.15%	-1.67	0.095
Firmicutes	Clostridia	Peptostreptococcales-Tissierellales	*Anaerovoracaceae*				0.10%	0.13%	0.03%	-1.32	0.187
Bacteroidota	Bacteroidia	Bacteroidales	*Prevotellaceae*				0.07%	0.08%	0.01%	-1.04	0.298
Bacteroidota	Bacteroidia	Bacteroidales	*Barnesiellaceae*				0.16%	0.01%	-0.15%	-3.29	0.001
Proteobacteria	Gammaproteobacteria	Burkholderiales	*Sutterellaceae*				0.36%	0.21%	-0.15%	-3.61	0.0003
Bacteroidota	Bacteroidia	Bacteroidales	*Marinifilaceae*				0.21%	0.04%	-0.17%	-3.85	0.0001
Desulfobacterota	Desulfovibrionia	Desulfovibrionales	*Desulfovibrionaceae*				0.21%	0.05%	-0.17%	-3.18	0.001
Firmicutes	Bacilli	Lactobacillales	*Streptococcaceae*				0.41%	0.19%	-0.22%	-0.24	0.810
Firmicutes	Clostridia	Oscillospirales	*Oscillospiraceae*				2.19%	1.94%	-0.25%	-0.83	0.407
Actinobacteriota	Coriobacteriia	Coriobacteriales	*Coriobacteriaceae*				2.10%	1.79%	-0.30%	-0.21	0.834
Bacteroidota	Bacteroidia	Bacteroidales	*Tannerellaceae*				0.97%	0.43%	-0.53%	-3.18	0.001
Bacteroidota	Bacteroidia	Bacteroidales	*Rikenellaceae*				1.24%	0.58%	-0.67%	-2.49	0.013
Firmicutes	Negativicutes	Acidaminococcales	*Acidaminococcaceae*				13.99%	3.12%	-10.88%	-2.24	0.025
Bacteroidota	Bacteroidia	Bacteroidales	*Bacteroidaceae*				27.58%	11.83%	-15.75%	-2.81	0.005
**Genus-level**											
Firmicutes	Clostridia	Oscillospirales	*Ruminococcaceae*	*Faecalibacterium*			6.57%	10.96%	4.39%	-3.36	0.001
Firmicutes	Clostridia	Lachnospirales	*Lachnospiraceae*	*Roseburia*			0.50%	2.06%	1.55%	-3.15	0.002
Firmicutes	Clostridia	Lachnospirales	*Lachnospiraceae*	*Lachnospiraceae NK4A136 group*			0.33%	1.70%	1.37%	-3.57	0.0004
Firmicutes	Clostridia	Oscillospirales	*Ruminococcaceae*	*Subdoligranulum*			0.60%	1.91%	1.32%	-3.22	0.001
Firmicutes	Clostridia	Lachnospirales	*Lachnospiraceae*	*Anaerostipes*			0.63%	1.94%	1.30%	-2.69	0.007
Firmicutes	Clostridia	Lachnospirales	*Lachnospiraceae*	*Agathobacter*			2.90%	4.08%	1.18%	-1.64	0.101
Firmicutes	Clostridia	Lachnospirales	*Lachnospiraceae*	*Blautia*			2.18%	3.30%	1.12%	-2.55	0.011
Firmicutes	Clostridia	Oscillospirales	*Ruminococcaceae*	*Ruminococcus*			0.80%	1.85%	1.05%	-2.20	0.028
Firmicutes	Clostridia	Monoglobales	*Monoglobaceae*	*Monoglobus*			0.53%	1.15%	0.62%	-3.53	0.0004
Firmicutes	Negativicutes	Veillonellales-Selenomonadales	*Veillonellaceae*	*Veillonella*			0.20%	0.76%	0.55%	-2.36	0.018
Firmicutes	Clostridia	Christensenellales	*Christensenellaceae*	*Christensenellaceae R-7 group*			0.23%	0.69%	0.46%	-3.45	0.001
Firmicutes	Clostridia	Lachnospirales	*Lachnospiraceae*	*Coprococcus*			0.79%	1.18%	0.39%	-1.12	0.263
Firmicutes	Clostridia	Oscillospirales	*Butyricicoccaceae*	*Butyricicoccus*			0.39%	0.75%	0.36%	-2.32	0.020
Actinobacteriota	Actinobacteria	Bifidobacteriales	*Bifidobacteriaceae*	*Bifidobacterium*			0.47%	0.74%	0.27%	-0.63	0.529
Actinobacteriota	Coriobacteriia	Coriobacteriales	*Eggerthellaceae*	*Adlercreutzia*			0.40%	0.65%	0.24%	-2.87	0.004
Firmicutes	Clostridia	Lachnospirales	*Lachnospiraceae*	*Dorea*			0.62%	0.74%	0.12%	-0.99	0.322
Firmicutes	Clostridia	Lachnospirales	*Lachnospiraceae*	*Lachnoclostridium*			0.65%	0.58%	-0.07%	-0.11	0.912
Firmicutes	Clostridia	Oscillospirales	*Oscillospiraceae*	*UCG-002*			0.65%	0.49%	-0.16%	-0.50	0.617
Actinobacteriota	Coriobacteriia	Coriobacteriales	*Coriobacteriaceae*	*Collinsella*			2.10%	1.79%	-0.30%	-0.21	0.834
Bacteroidota	Bacteroidia	Bacteroidales	*Tannerellaceae*	*Parabacteroides*			0.97%	0.43%	-0.53%	-3.18	0.001
Bacteroidota	Bacteroidia	Bacteroidales	*Rikenellaceae*	*Alistipes*			1.24%	0.53%	-0.72%	-2.49	0.013
Firmicutes	Negativicutes	Acidaminococcales	*Acidaminococcaceae*	*Phascolarctobacterium*			13.99%	3.12%	-10.88%	-2.18	0.029
Bacteroidota	Bacteroidia	Bacteroidales	*Bacteroidaceae*	*Bacteroides*			27.58%	11.83%	-15.75%	-2.81	0.005

**Species-level**											
Firmicutes	Clostridia	Oscillospirales	*Ruminococcaceae*	*Faecalibacterium*	*Faecalibacterium prausnitzii*		4.83%	7.47%	2.63%	-3.07	0.002
Firmicutes	Clostridia	Lachnospirales	*Lachnospiraceae*	*Blautia*	*Blautia obeum*		0.33%	0.80%	0.47%	-2.87	0.004
Firmicutes	Clostridia	Lachnospirales	*Lachnospiraceae*	*Anaerostipes*	*Anaerostipes hadrus*		0.35%	0.68%	0.33%	-2.48	0.013
Firmicutes	Clostridia	Lachnospirales	*Lachnospiraceae*	*Lachnospiraceae NK4A136 group*	*Lachnospiraceae NK4A136 group bacterium*		0.10%	0.35%	0.25%	-2.94	0.003
Firmicutes	Clostridia	Lachnospirales	*Lachnospiraceae*	*Roseburia*	*Roseburia inulinivorans*		0.09%	0.29%	0.20%	-0.71	0.478
Actinobacteriota	Coriobacteriia	Coriobacteriales	*Eggerthellaceae*	*Adlercreutzia*	*Adlercreutzia equolifaciens*		0.11%	0.29%	0.18%	-2.33	0.020
Firmicutes	Clostridia	Lachnospirales	*Lachnospiraceae*	*Roseburia*	*Roseburia hominis*		0.07%	0.22%	0.15%	-3.68	0.0002
Firmicutes	Clostridia	Lachnospirales	*Lachnospiraceae*	*Blautia*	*Blautia faecis*		0.21%	0.35%	0.13%	-3.56	0.0004
Firmicutes	Negativicutes	Veillonellales-Selenomonadales	*Veillonellaceae*	*Veillonella*	*Veillonella dispar*		0.06%	0.20%	0.13%	-2.13	0.033
Firmicutes	Clostridia	Oscillospirales	*Ruminococcaceae*	*Ruminococcus*	*Ruminococcus bromii*		0.06%	0.19%	0.13%	-0.37	0.711
Bacteroidota	Bacteroidia	Bacteroidales	*Bacteroidaceae*	*Bacteroides*	*Bacteroides thetaiotaomicron*		0.35%	0.46%	0.11%	-2.07	0.038
Actinobacteriota	Actinobacteria	Bifidobacteriales	*Bifidobacteriaceae*	*Bifidobacterium*	*Bifidobacterium longum*		0.09%	0.19%	0.10%	-1.31	0.190
Firmicutes	Clostridia	Lachnospirales	*Lachnospiraceae*	*Dorea*	*Dorea formicigenerans*		0.13%	0.19%	0.05%	-1.85	0.064
Firmicutes	Clostridia	Lachnospirales	*Lachnospiraceae*	*Dorea*	*Dorea longicatena*		0.43%	0.46%	0.03%	-0.40	0.689
Firmicutes	Clostridia	Lachnospirales	*Lachnospiraceae*	*Fusicatenibacter*	*Fusicatenibacter saccharivorans*		0.18%	0.22%	0.03%	-0.11	0.912
Bacteroidota	Bacteroidia	Bacteroidales	*Bacteroidaceae*	*Bacteroides*	*Bacteroides ovatus*		0.16%	0.11%	-0.05%	-0.07	0.944
Firmicutes	Clostridia	Lachnospirales	*Lachnospiraceae*	*Coprococcus*	*Coprococcus comes*		0.23%	0.13%	-0.09%	-2.01	0.044
Desulfobacterota	Desulfovibrionia	Desulfovibrionales	*Desulfovibrionaceae*	*Bilophila*	*Bilophila wadsworthia*		0.17%	0.03%	-0.13%	-3.15	0.002
Bacteroidota	Bacteroidia	Bacteroidales	*Bacteroidaceae*	*Bacteroides*	*Bacteroides caccae*		0.32%	0.17%	-0.15%	-2.87	0.004
Actinobacteriota	Coriobacteriia	Coriobacteriales	*Coriobacteriaceae*	*Collinsella*	*Collinsella aerofaciens*		1.93%	1.78%	-0.16%	-0.28	0.779
Bacteroidota	Bacteroidia	Bacteroidales	*Tannerellaceae*	*Parabacteroides*	*Parabacteroides merdae*		0.20%	0.01%	-0.19%	-2.10	0.036
Bacteroidota	Bacteroidia	Bacteroidales	*Tannerellaceae*	*Parabacteroides*	*Parabacteroides distasonis*		0.37%	0.12%	-0.25%	-3.16	0.002
Firmicutes	Clostridia	Lachnospirales	*Lachnospiraceae*	*Blautia*	*Blautia massiliensis*		0.50%	0.22%	-0.28%	-1.65	0.099
Bacteroidota	Bacteroidia	Bacteroidales	*Rikenellaceae*	*Alistipes*	*Alistipes putredinis*		0.73%	0.15%	-0.58%	-3.21	0.001
Bacteroidota	Bacteroidia	Bacteroidales	*Bacteroidaceae*	*Bacteroides*	*Bacteroides uniformis*		4.13%	2.16%	-1.97%	-0.93	0.352
Bacteroidota	Bacteroidia	Bacteroidales	*Bacteroidaceae*	*Bacteroides*	*Bacteroides vulgatus*		5.71%	2.71%	-3.00%	-2.99	0.003
Firmicutes	Negativicutes	Acidaminococcales	*Acidaminococcaceae*	*Phascolarctobacterium*	*Phascolarctobacterium faecium*		6.12%	0.47%	-5.65%	-0.87	0.384
**ASV-level**											
Firmicutes	Clostridia	Oscillospirales	*Ruminococcaceae*	*Faecalibacterium*	*Faecalibacterium prausnitzii*	13	0.50%	1.49%	0.98%	-3.39	0.001
Firmicutes	Clostridia	Monoglobales	*Monoglobaceae*	*Monoglobus*		16	0.37%	1.08%	0.72%	-3.53	0.0004
Firmicutes	Clostridia	Lachnospirales	*Lachnospiraceae*	*Blautia*	*Blautia obeum*	25	0.33%	0.80%	0.47%	-2.87	0.004
Firmicutes	Clostridia	Oscillospirales	*Ruminococcaceae*	*Faecalibacterium*		15	0.57%	1.00%	0.43%	-2.13	0.033
Firmicutes	Clostridia	Lachnospirales	*Lachnospiraceae*			20	0.48%	0.86%	0.37%	-3.51	0.0004
Firmicutes	Clostridia	Lachnospirales	*Lachnospiraceae*	*Anaerostipes*	*Anaerostipes hadrus*	32	0.21%	0.53%	0.33%	-2.38	0.017
Firmicutes	Clostridia	Lachnospirales	*Lachnospiraceae*			37	0.24%	0.52%	0.28%	-2.92	0.004
Firmicutes	Clostridia	Oscillospirales	*Ruminococcaceae*	*Faecalibacterium*	*Faecalibacterium prausnitzii*	2	1.57%	1.77%	0.20%	-1.83	0.067
Bacteroidota	Bacteroidia	Bacteroidales	*Bacteroidaceae*	*Bacteroides*	*Bacteroides thetaiotaomicron*	22	0.29%	0.46%	0.17%	-2.00	0.046
Firmicutes	Clostridia	Oscillospirales	*Ruminococcaceae*	*Subdoligranulum*		24	0.33%	0.50%	0.17%	-2.21	0.027
Firmicutes	Clostridia	Oscillospirales	*Ruminococcaceae*	*Subdoligranulum*		43	0.19%	0.35%	0.16%	-1.94	0.052
Firmicutes	Clostridia	Oscillospirales	*Ruminococcaceae*	*Faecalibacterium*	*Faecalibacterium prausnitzii*	10	1.18%	1.31%	0.12%	-0.12	0.904
Bacteroidota	Bacteroidia	Bacteroidales	*Bacteroidaceae*	*Bacteroides*	*Bacteroides uniformis*	4	0.21%	0.30%	0.08%	-1.36	0.174
Firmicutes	Clostridia	Lachnospirales	*Lachnospiraceae*	*Agathobacter*		5	2.07%	2.11%	0.04%	-1.89	0.059
Firmicutes	Clostridia	Lachnospirales	*Lachnospiraceae*	*Dorea*	*Dorea longicatena*	33	0.43%	0.46%	0.03%	-0.40	0.689
Firmicutes	Clostridia	Lachnospirales	*Lachnospiraceae*	*Blautia*		18	0.64%	0.54%	-0.10%	-0.32	0.749
Bacteroidota	Bacteroidia	Bacteroidales	*Bacteroidaceae*	*Bacteroides*		23	0.36%	0.24%	-0.12%	-1.37	0.171
Actinobacteriota	Coriobacteriia	Coriobacteriales	*Coriobacteriaceae*	*Collinsella*	*Collinsella aerofaciens*	6	1.93%	1.78%	-0.16%	-0.31	0.757
Firmicutes	Clostridia	Lachnospirales	*Lachnospiraceae*	*Blautia*	*Blautia massiliensis*	27	0.50%	0.22%	-0.28%	-1.65	0.099
Bacteroidota	Bacteroidia	Bacteroidales	*Bacteroidaceae*	*Bacteroides*	*Bacteroides uniformis*	7	1.06%	0.61%	-0.45%	-2.10	0.036
Bacteroidota	Bacteroidia	Bacteroidales	*Rikenellaceae*	*Alistipes*	*Alistipes putredinis*	40	0.73%	0.15%	-0.58%	-3.21	0.001
Bacteroidota	Bacteroidia	Bacteroidales	*Bacteroidaceae*	*Bacteroides*	*Bacteroides vulgatus*	8	1.28%	0.68%	-0.60%	-2.78	0.005
Bacteroidota	Bacteroidia	Bacteroidales	*Bacteroidaceae*	*Bacteroides*	*Bacteroides vulgatus*	3	2.97%	0.73%	-2.24%	-2.34	0.019
Firmicutes	Negativicutes	Acidaminococcales	*Acidaminococcaceae*	*Phascolarctobacterium*	*Phascolarctobacterium faecium*	1	6.12%	0.47%	-5.65%	-0.87	0.384

Supplementary Table S2. Taxonomic assignment (based on the Silva 138 database) and full sequences for those amplicon sequence variants (ASVs) that contributed significantly to the shifts in the gut microbiome across the two time points.

Supplementary Material.

ASV ID	Sequence	Taxonomic classification - Silva 138					
		Phylum	Class	Order	Family	Genus	Species
1	GCAGTGGGGAATCTTCCGCAATGGACGAAAGTCTGACGGAGCAACGCCGCGTGAGTGATGAAGGATTTCGGTCTGTAAAGCTCTGTTGTTTATGACGAACGTGCAGTGTGTGAACAATGCATTGCAATGACGGTAGTAAACGAGGAAGCCACGGCTAACTACGTGCCAGCAGCCGCGGTAATACGTAGGTGGCGAGCGTTGTCCGGAATTATTGGGCGTAAAGAGCATGTAGGCGGCTTAATAAGTCGAGCGTGAAAATGCGGGGCTCAACCCCGTATGGCGCTGGAAACTGTTAGGCTTGAGTGCAGGAGAGGAAAGGGGAATTCCCAGTGTAGCGGTGAAATGCGTAGATATTGGGAGGAACACCAGTGGCGAAGGCGCCT	Firmicutes	Negativicutes	Acidaminococcales	Acidaminococcaceae	Phascolarctobacterium	Phascolarctobacterium faecium
2	GCAGTGGGGAATATTGCACAATGGGGGAAACCCTGATGCAGCGACGCCGCGTGGAGGAAGAAGGTCTTCGGATTGTAAACTCCTGTTGTTGAGGAAGATAATGACGGTACTCAACAAGGAAGTGACGGCTAACTACGTGCCAGCAGCCGCGGTAAAACGTAGGTCACAAGCGTTGTCCGGAATTACTGGGTGTAAAGGGAGCGCAGGCGGGAAGACAAGTTGGAAGTGAAATCTATGGGCTCAACCCATAAACTGCTTTCAAAACTGTTTTTCTTGAGTAGTGCAGAGGTAGGCGGAATTCCCGGTGTAGCGGTGGAATGCGTAGATATCGGGAGGAACACCAGTGGCGAAGGCGGCCTACTGGGCACCAACTGACGCTGAGG	Firmicutes	Clostridia	Oscillospirales	Ruminococcaceae	Faecalibacterium	Faecalibacterium prausnitzii
3	GCAGTGAGGAATATTGGTCAATGGGCGAGAGCCTGAACCAGCCAAGTAGCGTGAAGGATGACTGCCCTATGGGTTGTAAACTTCTTTTATAAAGGAATAAAGTCGGGTATGGATACCCGTTTGCATGTACTTTATGAATAAGGATCGGCTAACTCCGTGCCAGCAGCCGCGGTAATACGGAGGATCCGAGCGTTATCCGGATTTATTGGGTTTAAAGGGAGCGTAGATGGATGTTTAAGTCAGTTGTGAAAGTTTGCGGCTCAACCGTAAAATTGCAGTTGATACTGGATATCTTGAGTGCAGTTGAGGCAGGCGGAATTCGTGGTGTAGCGGTGAAATGCTTAGATATCACGAAGAACTCCGATTGCGAAGGCAGCCTGCTA	Bacteroidota	Bacteroidia	Bacteroidales	Bacteroidaceae	Bacteroides	Bacteroides vulgatus
4	GCAGTGAGGAATATTGGTCAATGGACGAGAGTCTGAACCAGCCAAGTAGCGTGAAGGATGACTGCCCTATGGGTTGTAAACTTCTTTTATACGGGAATAAAGTGAGGCACGTGTGCCTTTTTGTATGTACCGTATGAATAAGGATCGGCTAACTCCGTGCCAGCAGCCGCGGTAATACGGAGGATCCGAGCGTTATCCGGATTTATTGGGTTTAAAGGGAGCGTAGGCGGACGCTTAAGTCAGTTGTGAAAGTTTGCGGCTCAACCGTAAAATTGCAGTTGATACTGGGTGTCTTGAGTACAGTAGAGGCAGGCGGAATTCGTGGTGTAGCGGTGAAATGCTTAGATATCACGAAGAACTCCGATTGCGAAGGCAGCTTGCTG	Bacteroidota	Bacteroidia	Bacteroidales	Bacteroidaceae	Bacteroides	Bacteroides uniformis
5	GCAGTGGGGAATATTGCACAATGGGCGAAAGCCTGATGCAGCGACGCCGCGTGAGCGAAGAAGTATTTCGGTATGTAAAGCTCTATCAGCAGGGAAGATAATGACGGTACCTGACTAAGAAGCACCGGCTAAATACGTGCCAGCAGCCGCGGTAATACGTATGGTGCAAGCGTTATCCGGATTTACTGGGTGTAAAGGGAGCGCAGGCGGTGCGGCAAGTCTGATGTGAAAGCCCGGGGCTCAACCCCGGTACTGCATTGGAAACTGTCGTACTAGAGTGTCGGAGGGGTAAGCGGAATTCCTAGTGTAGCGGTGAAATGCGTAGATATTAGGAGGAACACCAGTGGCGAAGGCGGCTTACTGGACGATAACTGACGCTGAGG	Firmicutes	Clostridia	Lachnospirales	Lachnospiraceae	Agathobacter	
6	GCAGTGGGGAATCTTGCGCAATGGGGGGAACCCTGACGCAGCGACGCCGCGTGCGGGACGGAGGCCTTCGGGTCGTAAACCGCTTTCAGCAGGGAAGAGTCAAGACTGTACCTGCAGAAGAAGCCCCGGCTAACTACGTGCCAGCAGCCGCGGTAATACGTAGGGGGCGAGCGTTATCCGGATTCATTGGGCGTAAAGCGCGCGTAGGCGGCCCGGCAGGCCGGGGGTCGAAGCGGGGGGCTCAACCCCCCGAAGCCCCCGGAACCTCCGCGGCTTGGGTCCGGTAGGGGAGGGTGGAACACCCGGTGTAGCGGTGGAATGCGCAGATATCGGGTGGAACACCGGTGGCGAAGGCGGCCCTCTGGGCCGAGACCGACGCTGAG	Actinobacteriota	Coriobacteriia	Coriobacteriales	Coriobacteriaceae	Collinsella	Collinsella aerofaciens
7	GCAGTGAGGAATATTGGTCAATGGACGAGAGTCTGAACCAGCCAAGTAGCGTGAAGGATGACTGCCCTATGGGTTGTAAACTTCTTTTATACGGGAATAAAGTGAGGCACGTGTGCCTTTTTGTATGTACCGTATGAATAAGGATCGGCTAACTCCGTGCCAGCAGCCGCGGTAATACGGAGGATCCGAGCGTTATCCGGATTTATTGGGTTTAAAGGGAGCGTAGGCGGACGCTTAAGTCAGTTGTGAAAGTTTGCGGCTCAACCGTAAAATTGCAGTTGATACTGGGTGTCTTGAGTACAGTAGAGGCAGGCGGAATTCGTGGTGTAGCGGTGAAATGCTTAGATATCACGAAGAACTCCGATTGCGAAGGCAGCCTGCTG	Bacteroidota	Bacteroidia	Bacteroidales	Bacteroidaceae	Bacteroides	Bacteroides uniformis
8	GCAGTGAGGAATATTGGTCAATGGGCGAGAGCCTGAACCAGCCAAGTAGCGTGAAGGATGACTGCCCTATGGGTTGTAAACTTCTTTTATAAAGGAATAAAGTCGGGTATGCATACCCGTTTGCATGTACTTTATGAATAAGGATCGGCTAACTCCGTGCCAGCAGCCGCGGTAATACGGAGGATCCGAGCGTTATCCGGATTTATTGGGTTTAAAGGGAGCGTAGATGGATGTTTAAGTCAGTTGTGAAAGTTTGCGGCTCAACCGTAAAATTGCAGTTGATACTGGATATCTTGAGTGCAGTTGAGGCAGGCGGAATTCGTGGTGTAGCGGTGAAATGCTTAGATATCACGAAGAACTCCGATTGCGAAGGCAGCCTGCTA	Bacteroidota	Bacteroidia	Bacteroidales	Bacteroidaceae	Bacteroides	Bacteroides vulgatus
10	GCAGTGGGGAATATTGCACAATGGGGGAAACCCTGATGCAGCGACGCCGCGTGGAGGAAGAAGGTCTTCGGATTGTAAACTCCTGTTGTTGAGGAAGATAATGACGGTACTCAACAAGGAAGTGACGGCTAACTACGTGCCAGCAGCCGCGGTAAAACGTAGGTCACAAGCGTTGTCCGGAATTACTGGGTGTAAAGGGAGCGCAGGCGGGAAGACAAGTTGGAAGTGAAATCCATGGGCTCAACCCATGAACTGCTTTCAAAACTGTTTTTCTTGAGTAGTGCAGAGGTAGGCGGAATTCCCGGTGTAGCGGTGGAATGCGTAGATATCGGGAGGAACACCAGTGGCGAAGGCGGCCTACTGGGCACCAACTGACGCTGAGG	Firmicutes	Clostridia	Oscillospirales	Ruminococcaceae	Faecalibacterium	Faecalibacterium prausnitzii
13	GCAGTGGGGAATATTGCACAATGGGGGAAACCCTGATGCAGCGACGCCGCGTGGAGGAAGAAGGTCTTCGGATTGTAAACTCCTGTTGTTGAGGAAGATAATGACGGTACTCAACAAGGAAGTGACGGCTAACTACGTGCCAGCAGCCGCGGTAAAACGTAGGTCACAAGCGTTGTCCGGAATTACTGGGTGTAAAGGGAGCGCAGGCGGGAGAACAAGTTGGAAGTGAAATCCATGGGCTCAACCCATGAACTGCTTTCAAAACTGTTTTTCTTGAGTAGTGCAGAGGTAGGCGGAATTCCCGGTGTAGCGGTGGAATGCGTAGATATCGGGAGGAACACCAGTGGCGAAGGCGGCCTACTGGGCACCAACTGACGCTGAGG	Firmicutes	Clostridia	Oscillospirales	Ruminococcaceae	Faecalibacterium	Faecalibacterium prausnitzii
15	GCAGTGGGGAATATTGCACAATGGGGGAAACCCTGATGCAGCGACGCCGCGTGGAGGAAGAAGGTCTTCGGATTGTAAACTCCTGTTGTTGGGGAAGATAATGACGGTACCCAACAAGGAAGTGACGGCTAACTACGTGCCAGCAGCCGCGGTAAAACGTAGGTCACAAGCGTTGTCCGGAATTACTGGGTGTAAAGGGAGCGCAGGCGGGAAGACAAGTTGGAAGTGAAATCTATGGGCTCAACCCATAAACTGCTTTCAAAACTGTTTTTCTTGAGTAGTGCAGAGGTAGGCGGAATTCCCGGTGTAGCGGTGGAATGCGTAGATATCGGGAGGAACACCAGTGGCGAAGGCGGCCTACTGGGCACCAACTGACGCTGAGG	Firmicutes	Clostridia	Oscillospirales	Ruminococcaceae	Faecalibacterium	
16	GCAGTGGGGAATATTGCGCAATGGGGGCAACCCTGACGCAGCAACGCCGCGTGCAGGAAGAAGGTCTTCGGATTGTAAACTGTTGTCGCAAGGGAAGAAGACAGTGACGGTACCTTGTGAGAAAGTCACGGCTAACTACGTGCCAGCAGCCGCGGTAATACGTAGGTGACAAGCGTTGTCCGGATTTACTGGGTGTAAAGGGCGCGTAGGCGGACTGTCAAGTCAGTCGTGAAATACCGGGGCTTAACCCCGGGGCTGCGATTGAAACTGACAGCCTTGAGTATCGGAGAGGAAAGCGGAATTCCTAGTGTAGCGGTGAAATGCGTAGATATTAGGAGGAACACCAGTGGCGAAGGCGGCTTTCTGGACGACAACTGACGCTG	Firmicutes	Clostridia	Monoglobales	Monoglobaceae	Monoglobus	
18	GCAGTGGGGAATATTGCACAATGGGGGAAACCCTGATGCAGCGACGCCGCGTGAAGGAAGAAGTATCTCGGTATGTAAACTTCTATCAGCAGGGAAGATAGTGACGGTACCTGACTAAGAAGCCCCGGCTAACTACGTGCCAGCAGCCGCGGTAATACGTAGGGGGCAAGCGTTATCCGGATTTACTGGGTGTAAAGGGAGCGTAGACGGTGTGGCAAGTCTGATGTGAAAGGCATGGGCTCAACCTGTGGACTGCATTGGAAACTGTCATACTTGAGTGCCGGAGGGGTAAGCGGAATTCCTAGTGTAGCGGTGAAATGCGTAGATATTAGGAGGAACACCAGTGGCGAAGGCGGCTTACTGGACGGTAACTGACGTTGAGG	Firmicutes	Clostridia	Lachnospirales	Lachnospiraceae	Blautia	
20	GCAGTGGGGAATATTGCACAATGGGGGAAACCCTGATGCAGCAACGCCGCGTGAGTGAAGAAGTATTTCGGTATGTAAAGCTCTATCAGCAGGGAAGATAATGACGGTACCTGACTAAGAAGCTCCGGCTAAATACGTGCCAGCAGCCGCGGTAATACGTATGGAGCAAGCGTTATCCGGATTTACTGGGTGTAAAGGGTGCGTAGGTGGCAGTGCAAGTCAGATGTGAAAGGCCGGGGCTCAACCCCGGAGCTGCATTTGAAACTGCTCGGCTAGAGTACAGGAGAGGCAGGCGGAATTCCTAGTGTAGCGGTGAAATGCGTAGATATTAGGAGGAACACCAGTGGCGAAGGCGGCCTGCTGGACTGTTACTGACACTGAGG	Firmicutes	Clostridia	Lachnospirales	Lachnospiraceae		
22	GCAGTGAGGAATATTGGTCAATGGGCGCAGGCCTGAACCAGCCAAGTAGCGTGAAGGATGACTGCCCTATGGGTTGTAAACTTCTTTTATATGGGAATAAAGTTTTCCACGTGTGGAATTTTGTATGTACCATATGAATAAGGATCGGCTAACTCCGTGCCAGCAGCCGCGGTAATACGGAGGATCCGAGCGTTATCCGGATTTATTGGGTTTAAAGGGAGCGTAGGTGGACAGTTAAGTCAGTTGTGAAAGTTTGCGGCTCAACCGTAAAATTGCAGTTGATACTGGCTGTCTTGAGTACAGTAGAGGTGGGCGGAATTCGTGGTGTAGCGGTGAAATGCTTAGATATCACGAAGAACTCCGATTGCGAAGGCAGCTCACTG	Bacteroidota	Bacteroidia	Bacteroidales	Bacteroidaceae	Bacteroides	Bacteroides thetaiotaomicron
23	GCAGTGAGGAATATTGGTCAATGGGCGAGAGCCTGAACCAGCCAAGTAGCGTGAAGGATGAAGGCTCTATGGGTCGTAAACTTCTTTTATATGGGAATAAAGTTTTCCACGTGTGGAATTTTGTATGTACCATATGAATAAGGATCGGCTAACTCCGTGCCAGCAGCCGCGGTAATACGGAGGATCCGAGCGTTATCCGGATTTATTGGGTTTAAAGGGAGCGTAGGTGGATTGTTAAGTCAGTTGTGAAAGTTTGCGGCTCAACCGTAAAATTGCAGTTGAAACTGGCAGTCTTGAGTACAGTAGAGGTGGGCGGAATTCGTGGTGTAGCGGTGAAATGCTTAGATATCACGAAGAACTCCGATTGCGAAGGCAGCTCACTA	Bacteroidota	Bacteroidia	Bacteroidales	Bacteroidaceae	Bacteroides	
24	GCAGTGGGGGATATTGCACAATGGGGGAAACCCTGATGCAGCGACGCCGCGTGGAGGAAGAAGGTTTTCGGATTGTAAACTCCTGTCGTTAGGGACGATAATGACGGTACCTAACAAGAAAGCACCGGCTAACTACGTGCCAGCAGCCGCGGTAAAACGTAGGGTGCAAGCGTTGTCCGGAATTACTGGGTGTAAAGGGAGCGCAGGCGGACCGGCAAGTTGGAAGTGAAAACTATGGGCTCAACCCATAAATTGCTTTCAAAACTGCTGGCCTTGAGTAGTGCAGAGGTAGGTGGAATTCCCGGTGTAGCGGTGGAATGCGTAGATATCGGGAGGAACACCAGTGGCGAAGGCGACCTACTGGGCACCAACTGACGCTGAGG	Firmicutes	Clostridia	Oscillospirales	Ruminococcaceae	Subdoligranulum	
25	GCAGTGGGGAATATTGCACAATGGGGGAAACCCTGATGCAGCGACGCCGCGTGAAGGAAGAAGTATCTCGGTATGTAAACTTCTATCAGCAGGGAAGATAGTGACGGTACCTGACTAAGAAGCCCCGGCTAACTACGTGCCAGCAGCCGCGGTAATACGTAGGGGGCAAGCGTTATCCGGATTTACTGGGTGTAAAGGGAGCGTAGACGGACTGGCAAGTCTGATGTGAAAGGCGGGGGCTCAACCCCTGGACTGCATTGGAAACTGTTAGTCTTGAGTGCCGGAGAGGTAAGCGGAATTCCTAGTGTAGCGGTGAAATGCGTAGATATTAGGAGGAACACCAGTGGCGAAGGCGGCTTACTGGACGGTAACTGACGTTGAGG	Firmicutes	Clostridia	Lachnospirales	Lachnospiraceae	Blautia	Blautia obeum
27	GCAGTGGGGAATATTGCACAATGGGGGAAACCCTGATGCAGCGACGCCGCGTGAAGGAAGAAGTATCTCGGTATGTAAACTTCTATCAGCAGGGAAGAAAATGACGGTACCTGACTAAGAAGCCCCGGCTAACTACGTGCCAGCAGCCGCGGTAATACGTAGGGGGCAAGCGTTATCCGGATTTACTGGGTGTAAAGGGAGCGTAGACGGATGGACAAGTCTGATGTGAAAGGCTGGGGCTCAACCCCGGGACTGCATTGGAAACTGCCCGTCTTGAGTGCCGGAGAGGTAAGCGGAATTCCTAGTGTAGCGGTGAAATGCGTAGATATTAGGAGGAACACCAGTGGCGAAGGCGGCTTACTGGACGGTAACTGACGTTGAGG	Firmicutes	Clostridia	Lachnospirales	Lachnospiraceae	Blautia	Blautia massiliensis
32	GCAGTGGGGAATATTGCACAATGGGGGAAACCCTGATGCAGCGACGCCGCGTGAGTGAAGAAGTATCTCGGTATGTAAAGCTCTATCAGCAGGGAAGAAAATGACGGTACCTGACTAAGAAGCCCCGGCTAACTACGTGCCAGCAGCCGCGGTAATACGTAGGGGGCAAGCGTTATCCGGAATTACTGGGTGTAAAGGGTGCGTAGGTGGTATGGCAAGTCAGAAGTGAAAACCCAGGGCTTAACTCTGGGACTGCTTTTGAAACTGTCAGACTGGAGTGCAGGAGAGGTAAGCGGAATTCCTAGTGTAGCGGTGAAATGCGTAGATATTAGGAGGAACATCAGTGGCGAAGGCGGCTTACTGGACTGAAACTGACACTGAGG	Firmicutes	Clostridia	Lachnospirales	Lachnospiraceae	Anaerostipes	Anaerostipes hadrus
33	GCAGTGGGGAATATTGCACAATGGAGGAAACTCTGATGCAGCGACGCCGCGTGAAGGATGAAGTATTTCGGTATGTAAACTTCTATCAGCAGGGAAGAAAATGACGGTACCTGACTAAGAAGCCCCGGCTAACTACGTGCCAGCAGCCGCGGTAATACGTAGGGGGCAAGCGTTATCCGGATTTACTGGGTGTAAAGGGAGCGTAGACGGCACGGCAAGCCAGATGTGAAAGCCCGGGGCTCAACCCCGGGACTGCATTTGGAACTGCTGAGCTAGAGTGTCGGAGAGGCAAGTGGAATTCCTAGTGTAGCGGTGAAATGCGTAGATATTAGGAGGAACACCAGTGGCGAAGGCGGCTTGCTGGACGATGACTGACGTTGAGG	Firmicutes	Clostridia	Lachnospirales	Lachnospiraceae	Dorea	Dorea longicatena
37	GCAGTGGGGAATATTGCACAATGGGGGAAACCCTGATGCAGCAACGCCGCGTGAGTGAAGAAGTATTTCGGTATGTAAAGCTCTATCAGCAGGGAAGATAATGACGGTACCTGACTAAGAAGCTCCGGCTAAATACGTGCCAGCAGCCGCGGTAATACGTATGGAGCAAGCGTTATCCGGATTTACTGGGTGTAAAGGGTGCGTAGGTGGCAGTGCAAGTCAGATGTGAAAGGCCGGGGCTCAACCCCGGAGCTGCATTTGAAACTGCATAGCTAGAGTACAGGAGAGGCAGGCGGAATTCCTAGTGTAGCGGTGAAATGCGTAGATATTAGGAGGAACACCAGTGGCGAAGGCGGCCTGCTGGACTGTTACTGACACTGAGG	Firmicutes	Clostridia	Lachnospirales	Lachnospiraceae		
40	GCAGTGAGGAATATTGGTCAATGGACGCAAGTCTGAACCAGCCATGCCGCGTGCAGGATGACGGCTCTATGAGTTGTAAACTGCTTTTGTACGAGGGTAAACGCAGATACGTGTATCTGTCTGAAAGTATCGTACGAATAAGGATCGGCTAACTCCGTGCCAGCAGCCGCGGTAATACGGAGGATTCAAGCGTTATCCGGATTTATTGGGTTTAAAGGGTGCGTAGGCGGTTTGATAAGTTAGAGGTGAAATTTCGGGGCTCAACCCTGAACGTGCCTCTAATACTGTTGAGCTAGAGAGTAGTTGCGGTAGGCGGAATGTATGGTGTAGCGGTGAAATGCTTAGAGATCATACAGAACACCGATTGCGAAGGCAGCTTACCA	Bacteroidota	Bacteroidia	Bacteroidales	Rikenellaceae	Alistipes	Alistipes putredinis
43	GCAGTGGGGGATATTGCACAATGGGGGAAACCCTGATGCAGCGACGCCGCGTGGAGGAAGAAGGTTTTCGGATTGTAAACTCCTGTCGTTAGGGACGATAATGACGGTACCTAACAAGAAAGCACCGGCTAACTACGTGCCAGCAGCCGCGGTAAAACGTAGGGTGCAAGCGTTGTCCGGAATTACTGGGTGTAAAGGGAGCGCAGGCGGGAAGACAAGTTGGAAGTGAAAACCATGGGCTCAACCCATGAATTGCTTTCAAAACTGTTTTTCTTGAGTAGTGCAGAGGTAGATGGAATTCCCGGTGTAGCGGTGGAATGCGTAGATATCGGGAGGAACACCAGTGGCGAAGGCGGTCTACTGGGCACCAACTGACGCTGAGG	Firmicutes	Clostridia	Oscillospirales	Ruminococcaceae	Subdoligranulum	

Supplementary Table S3. Amplicon sequence variants contributing to random forest model in PIME.

ASV ID in PIME	Full sequence of amplicon
Alistipes	GCAGTGAGGAATATTGGTCAATGGACGCAAGTCTGAACCAGCCATGCCGCGTGCAGGAAGACGGCTCTATGAGTTGTAAACTGCTTTTGTACGAGGGTAAACTCACCTACGTGTAGGTGACTGAAAGTATCGTACGAATAAGGATCGGCTAACTCCGTGCCAGCAGCCGCGGTAATACGGAGGATTCAAGCGTTATCCGGATTTATTGGGTTTAAAGGGTGCGTAGGCGGTTTGATAAGTTAGAGGTGAAATCCCGGGGCTTAACTCCGGAACTGCCTCTAATACTGTTAGACTAGAGAGTAGTTGCGGTAGGCGGAATGTATGGTGTAGCGGTGAAATGCTTAGAGATCATACAGAACACCGATTGCGAAGGCAGCTTACCA
Alistipes obesi	GCAGTGAGGAATATTGGTCAATGGACGGAAGTCTGAACCAGCCATGCCGCGTGCAGGAAGACGGCTCTATGAGTTGTAAACTGCTTTTGTACGAGGGTAAACGCAGATACGTGTATCTGCCTGAAAGTATCGTACGAATAAGGATCGGCTAACTCCGTGCCAGCAGCCGCGGTAATACGGAGGATCCAAGCGTTATCCGGATTTATTGGGTTTAAAGGGTGCGTAGGCGGTTTAGTAAGTCAGCGGTGAAATTTTGGTGCTTAACACCAAACGTGCCGTTGATACTGCTGGGCTAGAGAGTAGTTGCGGTAGGCGGAATGTATGGTGTAGCGGTGAAATGCTTAGAGATCATACAGAACACCGATTGCGAAGGCAGCTTACCA
Bacteroides ASV1	GCAGTGAGGAATATTGGTCAATGGACGAGAGTCTGAACCAGCCAAGTAGCGTGAAGGATGACTGCCCTATGGGTTGTAAACTTCTTTTATATGGGAATAAAGTATTCCACGTGTGGGATTTTGTATGTACCATATGAATAAGGATCGGCTAACTCCGTGCCAGCAGCCGCGGTAATACGGAGGATCCGAGCGTTATCCGGATTTATTGGGTTTAAAGGGAGCGTAGGTGGATTGTTAAGTCAGTTGTGAAAGTTTGCGGCTCAACCGTAAAATTGCAGTTGAAACTGGCAGTCTTGAGTACAGTAGAGGTGGGCGGAATTCGTGGTGTAGCGGTGAAATGCTTAGATATCACGAAGAACTCCGATTGCGAAGGCAGCTCACTA
Bacteroides ASV2	GCAGTGAGGAATATTGGTCAATGGGCGCAGGCCTGAACCAGCCAAGTAGCGTGAAGGATGACTGCCCTATGGGTTGTAAACTTCTTTTATAAAGGAATAAAGTCGGGTATGTATACCCGTTTGCATGTACTTTATGAATAAGGATCGGCTAACTCCGTGCCAGCAGCCGCGGTAATACGGAGGATCCGAGCGTTATCCGGATTTATTGGGTTTAAAGGGAGCGTAGATGGATGTTTAAGTCAGTTGTGAAAGTTTGCGGCTCAACCGTAAAATTGCAGTTGATACTGGATATCTTGAGTGCAGTTGAGGCAGGCGGAATTCGTGGTGTAGCGGTGAAATGCTTAGATATCACGAAGAACTCCGATTGCGAAGGCAGCCTGCTA
Bacteroides ASV3	GCAGTGAGGAATATTGGTCAATGGACGAGAGTCTGAACCAGCCAAGTAGCGTGAAGGATGACTGCCCTATGGGTTGTAAACTTCTTTTATATGGGAATAAAGTGAGCCACGTGTGGCTTTTTGTATGTACCATACGAATAAGGATCGGCTAACTCCGTGCCAGCAGCCGCGGTAATACGGAGGATCCGAGCGTTATCCGGATTTATTGGGTTTAAAGGGAGCGTAGGCGGACTATTAAGTCAGCTGTGAAAGTTTGCGGCTCAACCGTAAAATTGCAGTTGATACTGGTCGTCTTGAGTGCAGTAGAGGTAGGCGGAATTCGTGGTGTAGCGGTGAAATGCTTAGATATCACGAAGAACTCCGATTGCGAAGGCAGCTTACTG
Bacteroides ASV4	GCAGTGAGGAATATTGGTCAATGGGCGATGGCCTGAACCAGCCAAGTAGCGTGAAGGATGACTGCCCTATGGGTTGTAAACTTCTTTTATAAAGGAATAAAGTCGGGTATGCATACCCGTTTGCATGTACTTTATGAATAAGGATCGGCTAACTCCGTGCCAGCAGCCGCGGTAATACGGAGGATCCGAGCGTTATCCGGATTTATTGGGTTTAAAGGGAGCGTAGATGGATGTTTAAGTCAGTTGTGAAAGTTTGCGGCTCAACCGTAAAATTGCAGTTGATACTGGATGTCTTGAGTGCAGTTGAGGCAGGCGGAATTCGTGGTGTAGCGGTGAAATGCTTAGATATCACGAAGAACTCCGATTGCGAAGGCAGCCTGCTA
Bifidobacterium	GCAGTGGGGAATATTGCACAATGGGCGCAAGCCTGATGCAGCGACGCCGCGTGCGGGATGACGGCCTTCGGGTTGTAAACCGCTTTTGATCGGGAGCAAGCCTTCGGGTGAGTGTACCTTTCGAATAAGCACCGGCTAACTACGTGCCAGCAGCCGCGGTAATACGTAGGGTGCAAGCGTTATCCGGAATTATTGGGCGTAAAGGGCTCGTAGGCGGTTCGTCGCGTCCGGTGTGAAAGTCCATCGCTTAACGGTGGATCTGCGCCGGGTACGGGCGGGCTGGAGTGCGGTAGGGGAGACTGGAATTCCCGGTGTAACGGTGGAATGTGTAGATATCGGGAAGAACACCAATGGCGAAGGCAGGTCTCTGGGCCGTTACTGAC
Clostidrium sensu strico 1	GCAGTGGGGAATATTGCACAATGGGGGAAACCCTGATGCAGCAACGCCGCGTGAGTGATGACGGCCTTCGGGTTGTAAAGCTCTGTCTTCAGGGACGATAATGACGGTACCTGAGGAGGAAGCCACGGCTAACTACGTGCCAGCAGCCGCGGTAATACGTAGGTGGCGAGCGTTGTCCGGATTTACTGGGCGTAAAGGGAGCGTAGGCGGATTTTTAAGTGAGATGTGAAATACTCGGGCTTAACCTGAGTGCTGCATTTCAAACTGGAAGTCTAGAGTGCAGGAGAGGAGAAGGGAATTCCTAGTGTAGCGGTGAAATGCGTAGAGATTAGGAAGAACACCAGTGGCGAAGGCGCTTCTCTGGACTGTAACTGACGCTGAGG
Colidextribacter	GCAGTGGGGAATATTGGGCAATGGGCGCAAGCCTGACCCAGCAACGCCGCGTGAAGGAAGAAGGCTTTCGGGTTGTAAACTTCTTTTCTCAGGGACGAAGCAAGTGACGGTACCTGAGGAATAAGCCACGGCTAACTACGTGCCAGCAGCCGCGGTAATACGTAGGTGGCGAGCGTTATCCGGATTTACTGGGTGTAAAGGGCGTGTAGGCGGGACTGCAAGTCAGATGTGAAAACCATGGGCTCAACCTGTGGCCTGCATTTGAAACTGTAGTTCTTGAGTACTGGAGAGGCAGACGGAATTCCTAGTGTAGCGGTGAAATGCGTAGATATTAGGAGGAACACCAGTGGCGAAGGCGGTCTGCTGGACAGCAACTGACGCTG
Coprococcus	GCAGTGGGGAATATTGCACAATGGGGGAAACCCTGATGCAGCGACGCCGCGTGAGTGATGAAGTATTTCGGTATGTAAAACTCTATCAGCAGGGAAGATAATGACGGTACCTGACTAAGAAGCACCGGCTAAATACGTGCCAGCAGCCGCGGTAATACGTATGGTGCAAGCGTTATCCGGATTTACTGGGTGTAAAGGGTGCGTAGGTGGTATGGCAAGTCAGAAGTGAAAGGCTGGGGCTCAACCCCGGGACTGCTTTTGAAACTGTCAAACTAGAGTACAGGAGAGGAAAGCGGAATTCCTAGTGTAGCGGTGAAATGCGTAGATATTAGGAGGAACACCAGTGGCGAAGGCGGCTTTCTGGACTGAAACTGACACTGAGG
Eisenbergiella masilliensis	GCAGTGGGGAATATTGCACAATGGGGGAAACCCTGATGCAGCGACGCCGCGTGAGTGAAGAAGTATTTCGGTATGTAAAGCTCTATCAGCAGGGAAGAAAATGACGGTACCTGACTAAGAAGCCCCGGCTAACTACGTGCCAGCAGCCGCGGTAATACGTAGGGGGCAAGCGTTATCCGGATTTACTGGGTGTAAAGGGAGCGTAGACGGCATGACAAGCCAGATGTGAAAACCCAGGGCTCAACCCTGGGACTGCATTTGGAACTGCCAGGCTGGAGTGCAGGAGAGGTAAGCGGAATTCCTAGTGTAGCGGTGAAATGCGTAGATATTAGGAGGAACACCAGTGGCGAAGGCGGCTTACTGGACTGTAACTGACGTTGAGG
Faecalibacterium	GCAGTGGGGAATATTGCACAATGGGGGAAACCCTGATGCAGCGACGCCGCGTGGAGGAAGAAGGTCTTCGGATTGTAAACTCCTGTTGTTGAGGAAGATAATGACGGTACTCAACAAGGAAGTGACGGCTAACTACGTGCCAGCAGCCGCGGTAAAACGTAGGTCACAAGCGTTGTCCGGAATTACTGGGTGTAAAGGGAGCGCAGGCGGGCGATCAAGTTGGAAGTGAAATCCATGGGCTCAACCCATGAACTGCTTTCAAAACTGGTCGTCTTGAGTAGTGCAGAGGTAGGCGGAATTCCCGGTGTAGCGGTGGAATGCGTAGATATCGGGAGGAACACCAGTGGCGAAGGCGGCCTACTGGGCACCAACTGACGCTGAGG
Flavonifractor	GCAGTGGGGAATATTGGGCAATGGGCGCAAGCCTGACCCAGCAACGCCGCGTGAAGGAAGAAGGCTTTCGGGTTGTAAACTTCTTTTGTCAGGGACGAAGCAAGTGACGGTACCTGACGAATAAGCCACGGCTAACTACGTGCCAGCAGCCGCGGTAATACGTAGGTGGCAAGCGTTATCCGGATTTATTGGGTGTAAAGGGCGTGTAGGCGGGATTGCAAGTCAGATGTGAAAACTGGGGGCTCAACCTCCAGCCTGCATTTGAAACTGTAGTTCTTGAGTGTCGGAGAGGCAATCGGAATTCCGTGTGTAGCGGTGAAATGCGTAGATATACGGAGGAACACCAGTGGCGAAGGCGGATTGCTGGACGATAACTGACGCTG
Intestimonas butryciproducens	GCAGTGGGGAATATTGGGCAATGGGCGAAAGCCTGACCCAGCAACGCCGCGTGAAGGAAGAAGGCCCTCGGGTTGTAAACTTCTTTTGTCAGGGACGAAGCAAGTGACGGTACCTGACGAATAAGCCACGGCTAACTACGTGCCAGCAGCCGCGGTAATACGTAGGTGGCAAGCGTTATCCGGATTTACTGGGTGTAAAGGGCGTGTAGGCGGGAGTGCAAGTCAGATGTGAAAACTATGGGCTCAACCCATAGCCTGCATTTGAAACTGTACTTCTTGAGTGATGGAGAGGCAGGCGGAATTCCCTGTGTAGCGGTGAAATGCGTAGATATAGGGAGGAACACCAGTGGCGAAGGCGGCCTGCTGGACATTAACTGACGCTG
Lachnoclostridium ASV1	GCAGTGGGGAATATTGCACAATGGGCGAAAGCCTGATGCAGCGACGCCGCGTGAGTGAAGAAGTATTTCGGTATGTAAAGCTCTATCAGCAGGGAAGAAACTGACGGTACCTGACTAAGAAGCCCCGGCTAACTACGTGCCAGCAGCCGCGGTAATACGTAGGGGGCAAGCGTTATCCGGATTTACTGGGTGTAAAGGGAGCGTAGACGGCGAAGCAAGTCTGGAGTGAAAACCCAGGGCTCAACCCTGGGACTGCTTTGGAAACTGTTTTGCTAGAGTGTCGGAGAGGTAAGTGGAATTCCTAGTGTAGCGGTGAAATGCGTAGATATTAGGAGGAACACCAGTGGCGAAGGCGGCTTACTGGACGATAACTGACGTTGAGG
Lachnoclostridium ASV2	GCAGTGGGGAATATTGCACAATGGGGGAAACCCTGATGCAGCGACGCCGCGTGAAGGATGAAGTATTTCGGTATGTAAACTTCTATCAGCAGGGAAGAAGATGACGGTACCTGACTAAGAAGCCCCGGCTAACTACGTGCCAGCAGCCGCGGTAATACGTAGGGGGCAAGCGTTATCCGGATTTACTGGGTGTAAAGGGAGCGTAGACGGCGATGCAAGCCAGATGTGAAAGCCCGGGGCTCAACCCCGGGACTGCATTTGGAACTGCGTGGCTGGAGTGTCGGAGAGGCAGGCGGAATTCCTAGTGTAGCGGTGAAATGCGTAGATATTAGGAGGAACACCAGTGGCGAAGGCGGCCTGCTGGACGATGACTGACGTTGAGG
Lachnospira	GCAGTGGGGAATATTGCACAATGGAGGAAACTCTGATGCAGCGACGCCGCGTGAGTGAAGAAGTATTTCGGTATGTAAAGCTCTATCAGCAGGGAAGACAGTGACGGTACCTGACTAAGAAGCTCCGGCTAAATACGTGCCAGCAGCCGCGGTAATACGTATGGAGCAAGCGTTATCCGGATTTACTGGGTGTAAAGGGAGTGTAGGTGGTATCACAAGTCAGAAGTGAAAGCCCGGGGCTCAACCCCGGGACTGCTTTTGAAACTGTGGAACTGGAGTGCAGGAGAGGTAAGTGGAATTCCTAGTGTAGCGGTGAAATGCGTAGATATTAGGAGGAACACCAGTGGCGAAGGCGGCTTACTGGACTGTAACTGACACTGAGG
Lachnospiraceae FSC020 group	GCAGTGGGGAATATTGCACAATGGGGGAAACCCTGATGCAGCGACGCCGCGTGAGTGAAGAAGTATTTCGGTATGTAAAGCTCTATCAGCAGGGAAGAAAATGACGGTACCTGACTAAGAAGCCCCGGCTAACTACGTGCCAGCAGCCGCGGTAATACGTAGGGGGCAAGCGTTATCCGGATTTACTGGGTGTAAAGGGAGCGTAGACGGCTTTGCAAGTCTGACGTGAAAATCCGGGGCTCAACCCCGGAACTGCGTTGGAAACTGTGAGGCTTGAGTGCCGGAGAGGTAAGCGGAATTCCTAGTGTAGCGGTGAAATGCGTAGATATTAGGAGGAACACCAGTGGCGAAGGCGGCTTACTGGACGGCAACTGACGTTGAGG
Lachnospiraceae UC5-1-2-E3	GCAGTGGGGAATATTGCACAATGGGGGAAACCCTGATGCAGCGACGCCGCGTGAGTGATGAAGTATTTCGGTATGTAAAGCTCTATCAGCAGGGAAGAAAATGACGGTACCTGACTAAGAAGCCCCGGCTAACTACGTGCCAGCAGCCGCGGTAATACGTAGGGGGCAAGCGTTATCCGGATTTACTGGGTGTAAAGGGAGCGTAGACGGCTGTGCAAGTCTGGAGTGAAAGCCCGGGGCTCAACCCCGGGACTGCTTTGGAAACTGTACGGCTGGAGTGCTGGAGAGGCAAGCGGAATTCCTAGTGTAGCGGTGAAATGCGTAGATATTAGGAGGAACACCAGTGGCGAAGGCGGCTTGCTGGACAGTAACTGACGTTGAGG
Oscillospiraceae UCG-005	GCAGTGGGGAATATTGGGCAATGGGGGAAACCCTGACCCAGCAACGCCGCGTGAAGGAAGAAGGCTTTCGGGTTGTAAACTTCTTTTACCAGGGACGAAGAAAGTGACGGTACCTGGAGAAAAAGCCACGGCTAACTACGTGCCAGCAGCCGCGGTAATACGTAGGTGGCAAGCGTTGTCCGGATTTACTGGGTGTAAAGGGCGTGTAGGCGGAGATGCAAGTCAGATGTGAAATCCTCGGGCTTAACCCGGGAACTGCATTTGAAACTGTATCCCTTGAGTATCGGAGAGGCAGGCGGAATTCCTAGTGTAGCGGTGAAATGCGTAGATATTAGGAGGAACACCAGTGGCGAAGGCGGCCTGCTGGACGACAACTGACGCTG
Roseburia intestinalis	GCAGTGGGGAATATTGCACAATGGGGGAAACCCTGATGCAGCGACGCCGCGTGAGCGAAGAAGTATTTCGGTATGTAAAGCTCTATCAGCAGGGAAGAAGAAATGACGGTACCTGACTAAGAAGCACCGGCTAAATACGTGCCAGCAGCCGCGGTAATACGTATGGTGCAAGCGTTATCCGGATTTACTGGGTGTAAAGGGAGCGCAGGCGGTACGGCAAGTCTGATGTGAAAGCCCGGGGCTCAACCCCGGTACTGCATTGGAAACTGTCGGACTAGAGTGTCGGAGGGGTAAGTGGAATTCCTAGTGTAGCGGTGAAATGCGTAGATATTAGGAGGAACACCAGTGGCGAAGGCGGCTTACTGGACGATTACTGACGCTGA
Ruminococcaceae ASV1	GCAGTGAGGGATATTGGGCAATGGGGGCAACCCTGACCCAGCGACGCCGCGTGAGGGAAGACGGTCTTCGGATTGTAAACCTCTGTCTTTGGGGACGAAAAAGGACGGTACCCAAGGAGGAAGCTCCGGCTAACTACGTGCCAGCAGCCGCGGTAATACGTAGGGAGCGAGCGTTGTCCGGAATTACTGGGTGTAAAGGGAGCGTAGGCGGGAAGGCAAGTTGGATGTGAAAACTGTGGGCTCAACCTACAGACTGCATTCAAAACTGTTTTTCTTGAGTGAAGTAGAGGCAAGCGGAATTCCTAGTGTAGCGGTGAAATGCGTAGATATTAGGAGGAACACCAGTGGCGAAGGCGGCTTGCTGGGCTTTTACTGACGCTGAG
Ruminococcaceae ASV2	GCAGTGGGGGATATTGGTCAATGGGGGAAACCCTGAACCAGCAACGCCGCGTGAGGGAAGACGGCCTTCGGGTTGTAAACCTAAGTGATCAGGGACGAAGGAAGTGACGGTACCTGAAAAGCAAGCTCCGGCTAACTACGTGCCAGCAGCCGCGGTAATACGTAGGGAGCGAGCGTTGTCCGGATTTACTGGGTGTAAAGGGTGCGTAGGCGGGAAAGCAAGTCAGGTGTGAAATACCGGGGCTCAACTCCGGGGCTGCGCTTGAAACTGTTTTTCTTGAGTGAAGTAGAGGCAGGCGGAATTCCTAGTGTAGCGGTGGAATGCGTAGATATTAGGAGGAACACCAGTGGCGAAGGCGGCCTGCTGGGCTTTAACTGACGCTG
Ruminococcaceae incertae sedis	GCAGTGGGGGATATTGCACAATGGGGGAAACCCTGATGCAGCAACGCCGCGTGAGGGAAGAAGGTTTTCGGATTGTAAACCTCTGTTCTTAGTGACGATAATGACGGTAGCTAAGGAGAAAGCTCCGGCTAACTACGTGCCAGCAGCCGCGGTAATACGTAGGGAGCGAGCGTTGTCCGGATTTACTGGGTGTAAAGGGTGCGTAGGCGGCGAGGCAAGTCAGGCGTGAAATCTATGGGCTTAACCCATAAACTGCGCTTGAAACTGTCTTGCTTGAGTGAAGTAGAGGTAGGCGGAATTCCCGGTGTAGCGGTGAAATGCGTAGAGATCGGGAGGAACACCAGTGGCGAAGGCGGCCTACTGGGCTTTAACTGACGCTGAAG
Ruminococcus bromii	GCAGTGGGGGATATTGCGCAATGGGGGCAACCCTGACGCAGCAACGCCGCGTGAAGGATGAAGGTTTTCGGATTGTAAACTTCTTTTATTAAGGACGAAAAATGACGGTACTTAATGAATAAGCTCCGGCTAACTACGTGCCAGCAGCCGCGGTAATACGTAGGGAGCAAGCGTTGTCCGGATTTACTGGGTGTAAAGGGTGCGTAGGCGGCTTTGCAAGTCAGATGTGAAATCTATGGGCTCAACCCATAAACTGCATTTGAAACTGTAGAGCTTGAGTGAAGTAGAGGCAGGCGGAATTCCCCGTGTAGCGGTGAAATGCGTAGAGATGGGGAGGAACACCAGTGGCGAAGGCGGCCTGCTGGGCTTTAACTGACGCTGAG
Saccharimonadales	GCAGTGAGGAATATTTCACAATGAGCGAAAGCTTGATGGAGCAATGCCGCGTGCAGGATGAAGGCCCTCGGGTTGTAAACTGCTTTTATAAGAGAAGATTATGACGGTAACTTATGAATAAGGGACGGCTAACTACGTGCCAGCAGCCGCGGTCATACGTAGGTCCCGAGCGTTATCCGGAGTGACTGGGCGTAAAGAGTTGCGTAGGCGGTCAAGTTAGCGAGCGATGAAAACTATCGGCTCAACCGATAGCTTGTTGTTCGAACTGCTTGACTCGAGAGTATCAGAGGTCGCTGGAATTCCTAGTGTAGCAGTGAAATGCGTAGATATTAGGAAGAACACCGATGGCGTAGGCAGGCGACTGGGATATTTCTGACGCTAAG
Streptococcus	GCAGTAGGGAATCTTCGGCAATGGACGGAAGTCTGACCGAGCAACGCCGCGTGAGTGAAGAAGGTTTTCGGATCGTAAAGCTCTGTTGTAAGAGAAGAACGAGTGTGAGAGTGGAAAGTTCACACTGTGACGGTAACTTACCAGAAAGGGACGGCTAACTACGTGCCAGCAGCCGCGGTAATACGTAGGTCCCGAGCGTTATCCGGATTTATTGGGCGTAAAGCGAGCGCAGGCGGTTAGATAAGTCTGAAGTTAAAGGCTGTGGCTTAACCATAGTACGCTTTGGAAACTGTTTAACTTGAGTGCAGAAGGGGAGAGTGGAATTCCATGTGTAGCGGTGAAATGCGTAGATATATGGAGGAACACCGGTGGCGAAAGCGGCT
Tyzzerella	GCAGTGGGGAATATTGCACAATGGGGGAAACCCTGATGCAGCAACGCCGCGTGAAGGAAGAAGTATTTCGGTATGTAAACTTCTATCGACAGGGAAGAAACAAATGACGGTACCTGAATAAGAAGCACCGGCTAAATACGTGCCAGCAGCCGCGGTAATACGTATGGTGCAAGCGTTATCCGGATTTACTGGGTGTAAAGGGTGAGTAGGCGGTCATGCAAGTCATATGTGAAATGTCAGGGCTTAACCTTGGCGCTGCATAAGAAACTGTATGACTAGAGTGCAGGAGAGGTAAGCGGAATTCCTAGTGTAGCGGTGAAATGCGTAGATATTAGGAAGAACACCGGTGGCGAAGGCGGCTTACTGGACTGTTACTGACGCTG
Veillonella tobetsuensis	GCAGTGGGGAATCTTCCGCAATGGACGAAAGTCTGACGGAGCAACGCCGCGTGAGTGATGACGGCCTTCGGGTTGTAAAGCTCTGTTAATCGGGACGAAAGGCCTTCTTGCGAATAGTTAGAAGGATTGACGGTACCGGAATAGAAAGCCACGGCTAACTACGTGCCAGCAGCCGCGGTAATACGTAGGTGGCAAGCGTTGTCCGGAATTATTGGGCGTAAAGCGCGCGCAGGCGGATCAGTCAGTCTGTCTTAAAAGTTCGGGGCTTAACCCCGTGATGGGATGGAAACTGCTGATCTAGAGTATCGGAGAGGAAAGTGGAATTCCTAGTGTAGCGGTGAAATGCGTAGATATTAGGAAGAACACCAGTGGCGAAGGCGACT

Sequences for the top 29 amplicon sequence variants (ASVs) with the greatest contribution to the random forest model in PIME. Taxonomic assignments were derived from the Silva 138 database and used to derive a shortened ID for each ASV. ASV, amplicon sequence variant.

Supplementary Table S4. Amplicon sequence variants contributing to the classification of hsCRP subgroup at T2 at 40% prevalence.

ASV ID in PIME	Phylum	Class	Order	Family	Genus	Species	high	low	MeanDecreaseAccurac	MeanDecreaseGini	Average (High)	Average (low)	95% CI (high)	95% CI (low)	Mean difference
More abundant in high hsCRP															
Streptococcus	Firmicutes	Bacilli	Lactobacillal	Streptococca	*Streptococcus*		0.00457	0.00145	0.00278	0.09925	0.00030	0.00007	0.00035	0.00007	0.00023
Bacteroides ASV3	Bacteroidota	Bacteroidia	Bacteroidale	Bacteroidace	*Bacteroides*		0.00357	0.00447	0.00401	0.07685	0.00363	0.00192	0.00390	0.00176	0.00171
Alistipes	Bacteroidota	Bacteroidia	Bacteroidale	Rikenellacea	*Alistipes*		0.03483	0.02828	0.02739	0.65482	0.00796	0.00027	0.00876	0.00035	0.00769
Alistipes obesi	Bacteroidota	Bacteroidia	Bacteroidale	Rikenellacea	*Alistipes*	*obesi*	0.00367	0.00600	0.00484	0.18951	0.00028	0.00006	0.00016	0.00011	0.00022
Bacteroides ASV4	Bacteroidota	Bacteroidia	Bacteroidale	Bacteroidace	*Bacteroides*		0.01883	0.01352	0.01441	0.44277	0.02721	0.01120	0.01798	0.02157	0.01601
Bacteroides ASV2	Bacteroidota	Bacteroidia	Bacteroidale	Bacteroidace	*Bacteroides*		0.00290	0.00359	0.00297	0.07268	0.00919	0.00836	0.00870	0.00983	0.00083
Saccharimonadales	Patescibacte	Saccharimon	Saccharimonadales				0.00130	0.00450	0.00304	0.13270	0.00003	0.00000	0.00002	0.00000	0.00003
Ruminococcaceae ASV1	Firmicutes	Clostridia	Oscillospirale	Ruminococcaceae			0.00277	0.00407	0.00320	0.07723	0.00007	0.00006	0.00006	0.00008	0.00001
Lachnoclostridium ASV1	Firmicutes	Clostridia	Lachnospiral	Lachnospirac	*Lachnoclostridium*		0.00258	0.00279	0.00261	0.06951	0.00012	0.00006	0.00008	0.00007	0.00006
Tyzzerella	Firmicutes	Clostridia	Lachnospiral	Lachnospirac	*Tyzzerella*		0.00130	0.00450	0.00267	0.14640	0.00081	0.00066	0.00068	0.00083	0.00015
Lachnoclostridium ASV2	Firmicutes	Clostridia	Lachnospiral	Lachnospirac	*Lachnoclostridium*		0.00219	0.00319	0.00248	0.11688	0.00020	0.00009	0.00020	0.00016	0.00010
Roseburia intestinalis	Firmicutes	Clostridia	Lachnospiral	Lachnospirac	*Roseburia*	*intestinalis*	0.01390	0.01323	0.01180	0.33039	0.00192	0.00127	0.00119	0.00128	0.00065
Eisenbergiella masilliensis	Firmicutes	Clostridia	Lachnospiral	Lachnospirac	*Eisenbergiell*	*massiliensis*	0.00467	0.00195	0.00335	0.09737	0.00017	0.00011	0.00017	0.00008	0.00006
Lachnospiraceae FSC020 group	Firmicutes	Clostridia	Lachnospiral	Lachnospirac	*Lachnospiraceae_FCS020_*		0.00633	0.00635	0.00599	0.21928	0.00026	0.00003	0.00027	0.00003	0.00023
Coprococcus	Firmicutes	Clostridia	Lachnospiral	Lachnospirac	*Coprococcus*		0.00645	0.00500	0.00543	0.14413	0.00279	0.00193	0.00378	0.00371	0.00086
Lachnospiraceae UC5-1-2-E3	Firmicutes	Clostridia	Lachnospiral	Lachnospirac	*UC5-1-2E3*		0.00440	0.00507	0.00400	0.08860	0.00066	0.00009	0.00045	0.00011	0.00056
Colidextribacter	Firmicutes	Clostridia	Oscillospirale	Oscillospirac	*Colidextribacter*		0.00890	0.01227	0.01021	0.23003	0.00006	0.00002	0.00005	0.00002	0.00004
Flavonifractor	Firmicutes	Clostridia	Oscillospirale	Oscillospirac	*Flavonifractor*		0.00260	0.00482	0.00349	0.18913	0.00020	0.00012	0.00016	0.00012	0.00008
Veillonella tobetsuensis	Firmicutes	Negativicute	Veillonellales	Veillonellace	*Veillonella*	*tobetsuensis*	0.01133	0.00322	0.00590	0.22552	0.00163	0.00032	0.00241	0.00014	0.00132
Ruminococcaceae ASV2	Firmicutes	Clostridia	Oscillospirale	Ruminococcaceae			0.00320	0.00439	0.00372	0.09919	0.00006	0.00005	0.00005	0.00005	0.00001
More abundant in low hsCRP															
Bacteroides ASV1	Bacteroidota	Bacteroidia	Bacteroidale	Bacteroidace	*Bacteroides*		0.00873	0.01107	0.01005	0.32015	0.00483	0.00679	0.00315	0.01271	-0.00197
Lachnospira	Firmicutes	Clostridia	Lachnospiral	Lachnospirac	*Lachnospira*		0.00480	0.00107	0.00262	0.05840	0.00058	0.00145	0.00068	0.00087	-0.00088
Bifidobacterium	Actinobacter	Actinobacter	Bifidobacteri	Bifidobacteri	*Bifidobacterium*		0.01667	0.00742	0.01132	0.34913	0.00037	0.00253	0.00053	0.00317	-0.00216
Clostidrium sensu strico 1	Firmicutes	Clostridia	Clostridiales	Clostridiacea	*Clostridium_sensu_stricto_*		0.00773	0.00422	0.00528	0.17137	0.00007	0.00040	0.00012	0.00051	-0.00033
Faecalibacterium	Firmicutes	Clostridia	Oscillospirale	Ruminococca	*Faecalibacterium*		0.00717	0.00498	0.00548	0.16750	0.00123	0.00337	0.00147	0.00182	-0.00213
Intestimonas butryciproducens	Firmicutes	Clostridia	Oscillospirale	Oscillospirac	*Intestinimon*	*butyriciprodu*	0.00980	0.00701	0.00791	0.31560	0.00005	0.00032	0.00005	0.00054	-0.00026
Oscillospiraceae UCG-005	Firmicutes	Clostridia	Oscillospirale	Oscillospirac	*UCG-005*		0.00690	0.00277	0.00422	0.17837	0.00003	0.00023	0.00005	0.00016	-0.00020
Ruminococcaceae incertae sedis	Firmicutes	Clostridia	Oscillospirale	Ruminococca	*Incertae_Sedis*		0.00583	0.00124	0.00265	0.14429	0.00004	0.00011	0.00004	0.00010	-0.00007
Ruminococcus bromii	Firmicutes	Clostridia	Oscillospirale	Ruminococca	*Ruminococcu*	*bromii*	0.00510	0.00560	0.00524	0.21753	0.00135	0.00140	0.00095	0.00116	-0.00005

Statistics accompanying the PIME analysis including the top 29 amplicon sequence variants (ASVs) that contributed to the random forest classification of hsCRP clinical group at T2. Taxonomic assignment for the ASV is listed according to the Silva 138 database.

Supplementary Figure S1.

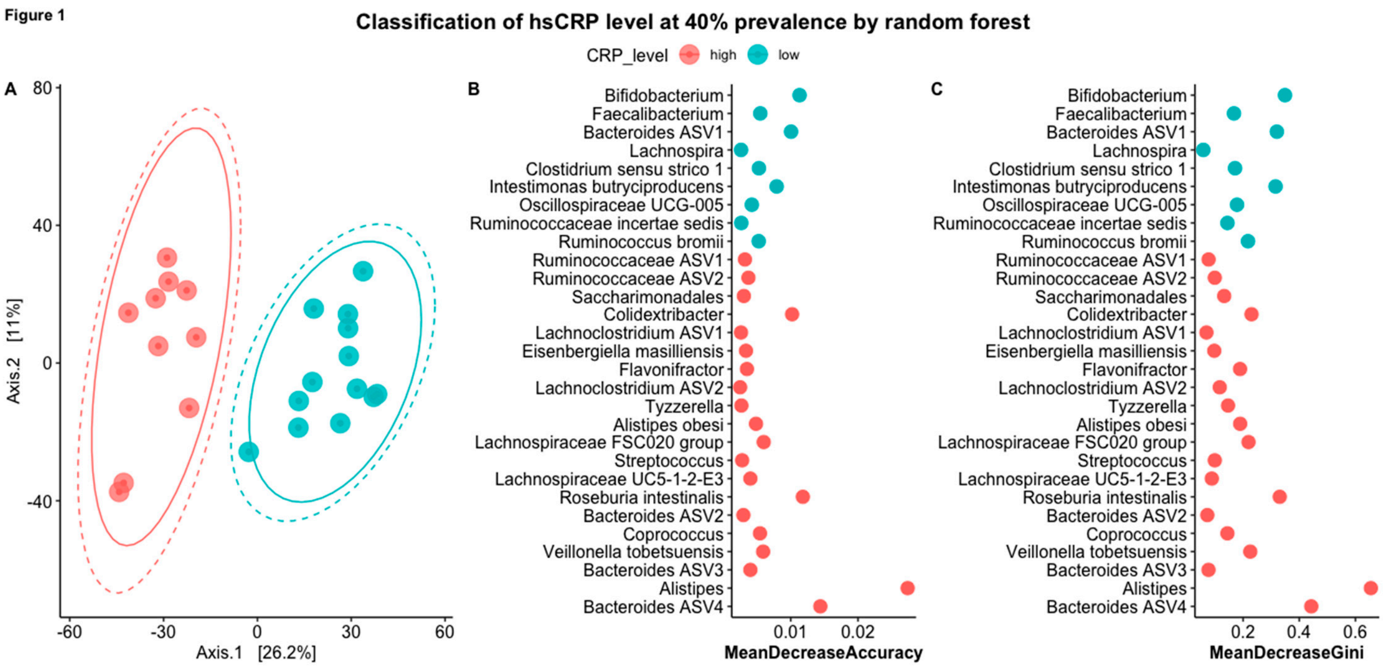

Distinct microbial communities present at 40% prevalence in low hsCRP and high hsCRP groups at T2. A) PCoA representation of the 240 microbial taxa that remained in the hsCRP high (N=10) and hsCRP low at T2 (N=13) groups at 40% prevalence (OOB error rate of 4.35%). Before filtering based on prevalence, there were a total 312 ASVs observed in at least five individuals (approximately 20% of the cohort), and 745 ASVs present in at least three individuals. Thus, this represents the microbial community after removing the least prevalent ASVs (124 ASVs removed) from those that were not present in at least 20% of the cohort. Ellipses represent the 95% confidence interval. CRP, C-reactive protein. B) Mean decrease accuracy for the top 29 ASVs in the classification, C) Mean Decrease Gini for the top 29 ASVs in the classification.

Supplementary Figure S2. PICRUSt functional profiles across time

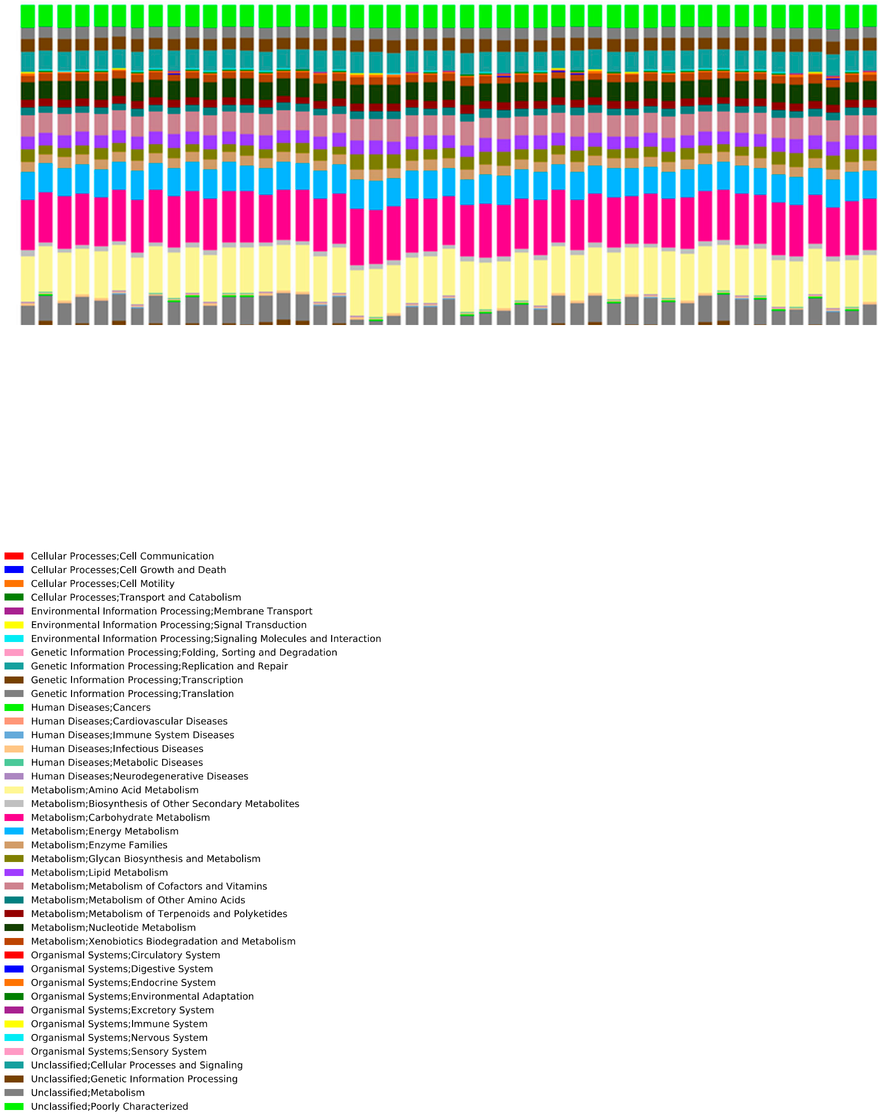

Supplement: Supplementary file 1 [file nutrients-13-03459-s001.zip › nutrients-1345315-supplementary.pdf]
